# A multi-modal deep learning framework with GAN-based fusion for enhanced landslide detection

**DOI:** 10.1371/journal.pone.0347324

**Published:** 2026-04-30

**Authors:** R. Srivats, Deepika Roselind Johnson, G. Logeswari, R. Saimirra, Muskaan Siddiqui, Abhiram Sharma

**Affiliations:** School of Computer Science and Engineering, Vellore Institute of Technology, Chennai Campus, Chennai, India; Sathyambama Institute of Science and Technology: Sathyabama Institute of Science and Technology (Deemed to be University), INDIA

## Abstract

The proposed work presents a hybrid deep learning framework that integrates four pre-trained Convolutional Neural Networks that include VGG16, DenseNet201, ResNet50 and InceptionV3. The pre-trained CNNs are combined with a GAN-based adversarial refinement module for accurate landslide detection and segmentation. Unlike traditional single-CNN or ensemble models, the proposed model performs multi-backbone feature fusion to accurately capture global level terrain context and fine-grained spatial details. The GAN component sharpens boundaries and suppresses noisy predictions through discriminator-guided refinement. The proposed system generates GIS-ready probability maps with confidence layers. They are also optimized for low-latency inference, making it suitable for rapid post-disaster decision support. The proposed work is evaluated on three benchmark datasets - CAS Landslide (high-resolution GF-2/UAV imagery), MS2LandsNet (medium-resolution Sentinel-2) and GDCLD (coseismic landslides). The proposed framework achieves F1-scores of 97.24%, 93.70% and 94.75% across the three datasets. These results correspond to improvements of 1.4 to 2.9% over fusion baselines and 4–7% over single-CNN models such as VGG16, DenseNet201,ResNet50 andInceptionV3. The results highlight consistent IoU gains and improved boundary delineation. The cross-dataset experiments further demonstrate strong generalization across varying resolutions, terrain types and triggering mechanisms. To our knowledge, this is the first landslide segmentation study to combine multi-backbone feature fusion with adversarial mask refinement in an operational monitoring context. The results confirm that the proposed framework delivers high accuracy, scalability and deployment readiness making.

## Introduction

Landslides are devastating natural disaster that results in life loss, infrastructure destruction and socio-economic impacts. Recent landslide incidents India (2024), Venezuela (2022), Madagascar (2021) and Indian Himalayas (2020) show that there has been an increase in frequency and severity of landslide occurrence. The increase in severity of these disasters has been attributed to rapid climate change, alarming rate of deforestation and quick urbanization. The conventional monitoring techniques include field surveys and remote sensing. However, these techniques are intensive, time consuming and are usually not adaptable for large-scale or remote areas. The traditional detection methods have a delayed response of 24–72 hours, time period considered crucial for saving lives and providing relief measures. These drawbacks necessitate automated, scalable and efficient landslide detection models to support early warning and mitigation. The adoption of deep learning techniques has transformed landslide detection and zone mapping. High-resolution images obtained from satellites and UAVs are analysed using analysed using Convolutional Neural Networks (CNNs). CNNs extract geospatial features and identify landslide-prone areas. Architectures such as VGG16 [1], DenseNet201 [2], ResNet50 [3] and InceptionV3 [4] show promising results. The improvement has been attributed to the model’s ability to learn hierarchical and multi-scale features from images. However, these models struggle due to coarse mask predictions, weak boundary prediction and reduced generalization due to dataset size limitations [5] and varied geo-spatial terrains.

To break those constraints, the Generative Adversarial Networks (GANs) have been developed as a contribution to image segmentation activities. GANs are capable of improving performance by refining the segmentation outputs by introducing a generator-discriminator adversarial architecture. The adversarial model reduces noise and mask outputs are realistic [6,7]. This is important especially in the detection of landslides where the boundary precision and generalization fine-grained. GANs have the capability to learn to produce and refine classifications on a pixel-level. They enhance both accuracy and robustness with limited training data. The proposed work introduces a new hybrid deep learning framework. The framework consists of a combination of various pre-trained CNN models and GAN-based adversarial refinement to detect landslides. In contrast to traditional methods that are based on individual CNN models or ensemble averaging technique. The proposed methodology employs multi-backbone fusion of VGG16, DenseNet201, ResNet50 and InceptionV3 to obtain both the general terrain characteristics and fine-grained features. The GAN element also improves precision in boundary detection by refining the boundaries. Recent segmentation approaches include CNN-Transformer hybrid models, diffusion models and Segment Anything Model (SAM) model. These models improve contextual understanding and generalization but involve high computational cost.

Models such as SE-YOLOv7 [8] and Conv-Transformer networks [9] show improved detection accuracy. However, they lack adversarial refinement mechanisms and feature fusion strategies. Many studies have employed hybrid CNN-GAN approaches with customized refinements when it comes to landslide detection. The customizations include pixel-wise segmentation and attention-based mask refinement. The novelty of the proposed approach lies in integrating CNN architecture with GAN-based adversarial mask refinement. This refinement achieves coarse-to-fine feature extraction with high-resolution boundary precision. This multi-backbone feature fusion with adversarial learning is rare in geospatial landslide detection. The multi-backbone feature fusion incorporated with adversarial learning is an unexplored area when it comes to landslide detection. The proposed model combines Binary Cross-Entropy (BCE), Intersection over Union (IoU), adversarial loss functions and stabilization techniques like Wasserstein loss with gradient penalty. The multi-objective loss function improves classification accuracy, region overlap and mask realism. The proposed model is considered as scalable, accurate and robust to support real-world landslide monitoring, rapid evacuation and disaster management initiatives. The proposed model is first-of-its-kind hybrid CNN-GAN framework exclusively used for landslide detection is introduced.

Three diverse and complementary datasets are used for evaluating the proposed model. The CAS Landslide [10] consists of 1,766 high-resolution images (512×512 pixels) obtained from GF‑2 satellite and UAV surveys obtained in Sichuan Province, China. The Sichuan Province is an area characterized by rugged topography, steep terrain and landslide triggered by monsoon. The MS2LandsNet dataset [11] consists of medium-resolution images obtained from Sentinel‑2 sourced from Luding and Jiuzhaigou, China. The dataset from these areas are used for introducing seasonal variations and for broad generalization. The Globally Distributed Coseismic Landslide Dataset (GDCLD) [12] consists of high-resolution remote sensing images of coseismic events obtained from multiple locations (Wenchuan and Ludian). The dataset is sourced from PlanetScope, Gaofen-6, Map World and UAV data across diverse geographic and geological settings. The dataset is suitable for application of deep learning models for landslide mapping evaluation. The proposed approach achieves outstanding performance (97.24%) that surpasses many state-of-the-art methods.

The contributions of the proposed work are as follows:

- Propose a novel hybrid CNN-GAN segmentation framework with multi-backbone feature fusion of VGG16, DenseNet201, ResNet50 and InceptionV3 through feature-level fusion. It is combined with GAN-based adversarial refinement module to enhance segmentation.
- Build a sensor-agnostic preprocessing pipeline with documented splits and label QA, reporting calibrated F1-score, Intersection over Union (IoU), Precision – Accuracy and Matthews Correlation Coefficient (MCC).
- The proposed model achieves F1-scores on CAS Landslide (97.24%), MS2LandsNet (93.70%) and GDCLD datasets (94.75%). It outperforms fusion baselines by 1.4 to 2.9% and single-CNN models by 4–7% with consistent improvements in IoU. The model achieves an inference of 88 ms per images which enables near real-time detection and reduces delay in response.
- The proposed framework demonstrates strong cross-dataset generalization across varying resolutions (high versus medium resolution), trigger types (rainfall versus coseismic) and varied geographic conditions. It was observed to consistently maintain F1-scores above 93% across all three datasets.

The reminder of the paper is structured as follows, Related Work section focuses on the literature survey conducted on CNNs to detect landslides. The Proposed methodology section provides model design, components and their construction. The results and discussion section describes the datasets used for evaluation, experimentation and evaluation of the proposed framework with baseline and benchmark models. It provides quantitative and qualitative analysis with baseline methods along with performance metrics. Finally, conclusion section highlights the concluding remarks and provides an overview of the potential directions for future research.

## Related works

Landslide detection and monitoring have been experiencing a swift change with the growing availability of multisource remote sensing data and improvements in deep learning. Conventional approaches are reliant on manual interpretation, heuristics or machine learning-based classifiers which improved mapping capability of baseline models. They had limitations with respect to application, scalability and sensitivity to change in illumination and environmental noise. These drawbacks facilitated the requirement for automated deep-learning models to learn discriminative patterns from images.

Zhang et al. applied EfficientDet for localizing landslide features using labelled datasets that were created in LabelMe [13,14]. It showed promising results in terms of precision and efficiency of the training process. Similarly, Bui et al. combined convolutional neural networks and Hue-Bi-dimensional Empirical Mode Decomposition algorithm to increase the robustness in varying lighting situations with a maximum accuracy of 96% [15]. Even though object-detection models can detect landslide geometry with quick localization, the bounding-box approach limits the availability of detecting irregular landslide geometry.

As a result, CNN architectures based on segmentation have become popular. Chen et al. proposed BisDeNet, which is based on BiSeNet but incorporates DenseNet to enhance the representation of features with fewer parameters [16]. Ghorbanzadeh et al. further showed the advantage of combining pixel-based ResU-Net models and object-based image analysis (OBIA). The approach was an integration of ResU-Net-OBIA pipeline and outperformed many methods [17]. Meanwhile, an investigation by Nava et al. investigated the performance of CNN using both optical and SAR images after an earthquake in Hokkaido and demonstrated a performance of nearly 95% in the presence of adverse weather conditions [18] where network models (pixel-level) display fidelity in shape and improve robustness. A parallel work was made comparing mainstream deep detectors like SSD, YOLOv3 and Faster R-CNN that analysed trade-off between accuracy and speed [7,19]. While Faster R-CNN got a higher precision, regression-based detectors were faster.

The detection reliability can be improved by using hybrid remote-sensing strategies. Chandra et al. focussed on the need for multispectral, LiDAR and SAR integration with ML algorithms such as random forest and logistic regression to overcome the limitations of single sensor systems [20]. Similarly, hybrid learning environments based on XGBoost and rough set theory showed higher levels of prediction flexibility in complex terrain [21]. Studies conducted by Al-Najjar et al. supported the fact that the consideration of conditioning factors like slope and altitude improves the accuracy of susceptibility mapping and model interpretation [22]. These studies were found to emphasize on the usefulness of data fusion strategies in diverse environments. Subsequently, a lot of progress in designing more efficient deep-learning architectures has been achieved. Song et al. proposed an SBConv Enhanced U-Net with better feature extraction for Multi-Source Imagery [7]. It is a light network with MS2LandsNet to incorporate multiscale fusion and channel attention to support effective landslide recognition even at medium resolution [23]. Auflic et al., Casagli et al. and other authors emphasized the operational need for scalable models that are able to support monitoring systems in national geological agencies [24–27].

SE-YOLOv7, an attention-driven detector integrates squeeze-and-excitation using advanced loss functions to refine boundaries and minimizes false positives [28]. Meanwhile, the CNN/Transformer hybrid networks proposed by Yu et al. and Zhou et al. overcome the shortcomings of CNNs in modelling long range dependencies which results in more contextual reasoning and edge precision [29,30]. Transfer learning from foundation models has also been helpful. For example, TransLandSeg exploits SAM but dramatically downsamples trainable parameters without hurting the segmentation fidelity. [31]. Iterative contrastive learning schemes have shown better results on old-landslide segmentation [32]. LMHLD [33] provides a comprehensive testbed that can be accessed for multiple hazard regions. The datasets help researchers quantify generalization and help in knowledge transfer beyond isolated case studies.

Recent deep-learning techniques provide architectural support and customizations. The models employ stacked encoder-decoders, adversarial feedback and progressive optimization [34–36]. These advanced models enhance feature representation and generalization. Although these strategies were initially developed for biomedical or tasks of sequence analysis, they are similar to the objectives of landslide segmentation, particularly in terms of balancing robustness and realism of output. Recent progress in landslide segmentation has taken advantage of foundation models such as the Segment Anything Model (SAM) [37,38], diffusion-based generative models [39–41] and hybrid CNN-Transformer models [42–45] to enhance the contextual understanding and segmentation accuracy. These approaches fail to include adversarial refinement and multi-backbone fusion for precise boundary detection.

The literature review provides overlapping insights on various models. It can be noted that detection-based models and CNNs provide improved segmentation performance but face high computational cost. These lightweight architectures improve scalability but are constrained in terms of robustness when it comes to complex terrains. Hybrid models considering attention and transformer enhance the contextual learning, and multi-sensor fusion enhances the resilience against the environmental variability. However, very few approaches have integrated multi-backbone feature fusion, adversarial refinement and efficiency-aware design in one unified framework. In order to overcome these disadvantage, a hybrid Multi-CNN and GAN multi-backbone feature fusion model is proposed with pre-trained backbones and incorporates adversarial refinement module to enhance structural coherence and boundary precision for landslide detection.

## Proposed methodology

**Fig 1:**
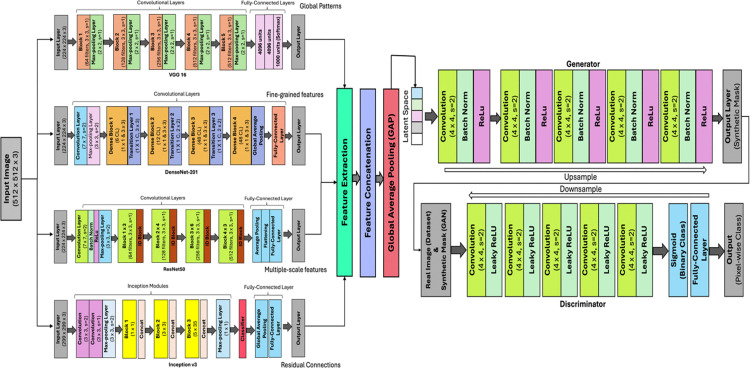
Architecture of the proposed multi-modal deep learning framework with GAN-based fusion for landslide detection.

The proposed methodology (Fig 1) is structured to effectively perform landslide detection using deep learning. Initially, the dataset is pre-processed where raw images and their corresponding masks are transformed for standardization and diversity. After preprocessing, a combined deep learning model is implemented to harnesses the strengths of pre-trained architectures such as VGG16 and DenseNet201. VGG16 and DenseNet201 are chosen for complementary feature extraction that captures both high-level structures and intricate details in landslide imagery. This unified framework is used for pixel-level classification of landslides and too enhance detection accuracy. The overall of the unified framework is shown in Fig 1. The processing pipeline consists of four sequential stages:

i Data preprocessing and augmentations.
ii Feature extraction using multiple CNN backbones.
iii Feature fusion using channel-wise concatenation.
iv GAN-based adversarial refinement for segmentation.

### Data pre-processing

The pre-processing pipeline is initialized with the exporting of ‘LandslideDataset’ class. The class loads raw landslide images and their corresponding segmentation masks for further processing. It groups the images and segmentation mask and further transformed for model training. These masks consist of pixel-wise annotations of landslide regions which serves as ground truth for supervised learning. This maintains the data in a uniform format, enabling the model to learn effective patterns without the need to concern itself with formatting.


$$D=\{\left(I_{\text{1}},M_{\text{1}}\right),\;\left(I_{\text{2}},M_{\text{2}}\right),\;\ldots ,\;\left(I_{n},M_{n}\right)\}$$ (1)

where $I_{i}$ represents the i^th^ image, $M_{i}$ is the corresponding mask for image $I_{i}$ and n is number of images. Once the images and masks are loaded, they undergo a resizing process where both are scaled to a fixed dimension of 256×256 pixels using the transformation function. Resizing is necessary to ensure that all images and masks have the same dimension of 256×256 pixels. Given an original image with dimensions H×W and its corresponding mask, both are resized to a uniform dimension 256×256 pixels. If the original image size is H×W, the resizing process is represented as,


$$I_{resized}=Resize(I,\;(\text{2}\text{5}\text{6},\;\text{2}\text{5}\text{6}))$$ (2)

$$M_{resized}=Resize(M,\;(\text{2}\text{5}\text{6},\;\text{2}\text{5}\text{6}))$$ (3)

where $I_{resized}$ and $M_{resized}$ are the resized versions of the image and the mask, respectively. The transformation Resize(I,(256,256)) is done using the transformation function transforms.Resize((256,256)). Resizing all images and masks to 256×256 pixels provide uniformity, which is necessary for CNNs that only work with fixed input sizes. Uniformity makes learning easier, optimizes memory usage and leads to stable training and improved generalization to unseen data. After resizing, the images are converted from Python Imaging Library (PIL) format into PyTorch tensors using the transforms.ToTensor() function. The images are then converted to PyTorch tensors, which involves scaling pixel values from their original range of [0,255] to [0,1]. The transformation is given by,


$$I_{tensor}=\frac{I_{resized}}{\text{2}\text{5}\text{5}}$$ (4)

Equation (4) resizes the images from PIL format to PyTorch tensors, normalizing the pixel values from [0, 255] to [0, 1]. Although binary masks (as 0 or 1) are not typically scaled, this operation is done to maintain compatibility with PyTorch models. Pixel value normalization enhances training stability, minimizes large gradients and facilitates faster convergence.

Normalization standardizes the pixel values of images by transforming them to have a mean of zero and a variance of one, based on the dataset’s calculated mean and standard deviation. This process ensures that the data aligns with the input distribution expected by pre-trained models such as VGG16 and DenseNet201. The formula for normalization is,


$$I_{norm}=\frac{I_{tensor}-\mu }{\sigma }$$ (5)

where $I_{norm}$ is the normalized image, μ is the mean pixel intensity value calculated over the entire dataset and σ is the standard deviation of the pixel intensity values in the dataset. Normalization ensures that the input data distribution has a mean of 0 and a standard deviation of 1, which helps stabilize the training process and speeds up convergence.

Normalization aligns pre-trained models like VGG16 and DenseNet’s pixel distribution in the dataset to adhere to those trained on datasets such as ImageNet. Normalizing images by mean and standard deviation of pixel values stabilizes training, improves model stability and minimizes vanishing or exploding gradients. To enhance the model’s robustness, data augmentation strategies are applied to enhance the variety of the dataset. In the landslide dataset, operations such as resizing, grayscale conversion, flipping and rotation are applied, with further resizing being applied to mimic different image scales. Given an image I resized to 256×256, further resizing to other scales $s_{\text{1}},\;s_{\text{2}},\;\ldots ,\;s_{n}\;$ can be done as,


$$I_{aug-resize}=Resize\left(I,(s,s)\right),\;s\;\epsilon \;\{s_{\text{1}},\;s_{\text{2}},\;\ldots ,\;s_{n}\}$$ (6)

In addition to the initial resizing 256×256 pixels, further resizing can be applied at different scales to simulate variations in image size that the model might encounter in real-world scenarios. Grayscale conversion converts the RGB image into a single channel grayscale image using the weighted sum of the RGB channels. This transformation is represented as,


$$I_{gray}=\text{0}.\text{2}\text{9}\text{9}\cdot R+\text{0}.\text{5}\text{8}\text{7}\cdot G+\text{0}.\text{1}\text{1}\text{4}\cdot B$$ (7)

where R, G and B are the red, green and blue colour channels respectively and $I_{gray}$ is the grayscale image. Converting the images to grayscale degrades the complexity of the input by concentrating on the variations in intensity, which are important in identifying the presence of a landslide in some environments where colour information may not be as important. Random horizontal and vertical flipping of the images provides spatial variability, so that the model could understand the landslide from different orientations. Random flipping (horizontally or vertically), can be achieved by applying transformation function F to the original image.


$$I_{flipped}=F(I)$$ (8)

where angle correspond to the seven rotations ranging from ${\text{0}}^{\circ }$ to ${\text{2}\text{7}}^{{\text{0}}^{\circ }}$ is applied to each image present in the dataset.

These augmentation strategies make the model better capable of detecting a variety of landslide patterns and configurations, and as such enhance the generalization capability of the model. By training the model on augmented dataset, overfitting likelihood is reduced with respect to specific landslide patterns. The transformations that are performed on each image I in the dataset can be represented as,


$$I_{aug}=\left\{Resize,\;Grayscale,Flip,R_{\theta }\right\}(I)$$ (9)

where $I_{aug}$ is the version of the original image after all the augmentation techniques are applied. The combination of resizing, normalization and augmentation does not only improve the quality of the dataset but also increases the generalization capacity of the model. Overfitting leads the model to perform poorly on landslide detection. The model’s performance is made robust by making it invariant to changes in orientation, size and lighting conditions by training with various transformations of the same image during training process.

### Model selection and combination

The networks VGG16, DenseNet201, ResNet50 and InceptionV3 were chosen because of their complementary features extraction capabilities. The hierarchical global pattern is captured by VGG16 while feature and fine-grained detail extraction is performed by DenseNet201. ResNet50 makes deep residual learning for good semantic representation and InceptionV3 offers multi-scale feature analysis. The combined model captures diverse spatial and contextual features ignored by single or few backbone architectures. It provides a good balance between feature diversity, training stability and small differences in landslide regions.

#### VGG16.

VGG16, a CNN extracts hierarchical features that captures global patterns from an input image. The model consists of multiple convolutional layers (CLs), each followed by ReLU (Rectified Linear Unit) activations and max-pooling layers. In VGG16, the convolutional layers are responsible for learning hierarchical patterns. The feature map (FM) from a CL for an input image I is represented as,


$$F_{l}(I)=W_{l}*I+b_{l}$$ (10)

where $F_{l}(I)$ is the FM at layer l, $W_{l}$ represents the learned weights (filter), I is the input image or FM from the previous layer, * denotes the convolution operation and $b_{l}$ is bias term. After each CL, a ReLU activation function is applied to introduce non-linearity.


$$A_{l}(F)=\text{max}(\text{0},F_{l}(I))$$ (11)

where $A_{l}(F)$ is the output after the activation function is applied and $F_{l}(I)$ is the feature map from the convolutional layer. VGG16 also applies max pooling after certain layers to downsample the feature maps, which reduces the spatial dimensions while preserving the most important information.


$$P\left(F_{l}(I)\right)=\underset{\left(m,n\right)}{\text{max}}\left(F_{l}(I)\right)$$ (12)

where $P(F_{l}(I))$ is the pooled feature map, (m,n) represents the window size over which the maximum operation is performed. The combination of convolution, ReLU activation and max pooling allow VGG16 to extract high level and broad structural patterns that are necessary for identifying general regions of interest, similar to landslide areas, in the input image.

#### DenseNet201.

DenseNet201 captures fine details via densely connected layers. DenseNet has a unique architecture where each layer gets the FMs of all preceding layers and the network is able to retain information and learn subtle differences in the input image. Each layer l obtains feature maps from the previous layers and represented as,


$$F_{l}=H_{l}(\left[F_{\text{0}},F_{\text{1}},\ldots ,F_{l-\text{1}}\right])$$ (13)

where $H_{l}$ is the transformation function applied at layer l (composed of convolution and activation) and $\left[F_{\text{0}},F_{\text{1}},\ldots ,F_{l-\text{1}}\right]$ is the concatenated FM from all earlier layers. The dense connectivity encourages feature reuse and helps flow of gradients during backpropagation which helps the model more detailed and more complex features, especially useful for distinguishing between subtle landslide areas and background. DenseNet has bottleneck layers which reduce the number of feature maps prior to performing the convolution operation This is accomplished by making use of a 1×1 convolution.


$$F_{bottle}=W_{\text{1}\times \text{1}}*F_{prev}$$ (14)

where $F_{bottle}$ is the output of the bottleneck layer and $W_{\text{1}\times \text{1}}$ is the 1×1 convolution filter that is applied to the feature maps $F_{prev}$. Bottleneck layers increase computational efficiency while preserving the key information necessary for the detection of fine-grained features.

#### ResNet50.

The ResNet50 module is an important part to derive hierarchical and residual features from input, solving the vanishing gradient problem with residual connections. This capability is especially critical for landslide detection, where fine-grained spatial features and global patterns are captured. ResNet50 consists of two main structures that include convolutional- and identity blocks. The convolutional block containing a series of 1×1, 3×3 and 1×1 convolution followed by Batch Normalization (BN) combined with ReLU activation function The 1×1 convolutions are intended to downsize and revive dimensionality and the 3×3 convolution is focused on capturing spatial dependencies, such as soil erosion, rock texture and vegetation displacement. In contrast, identity block avoids certain convolutions, in order to ensure computational efficiency. The residual connection is key to ResNet50’s architecture and allowing model to learn residual mappings. The output y of a residual connection is expressed as:


$$y=F\left(x\left\{W_{i}\right\}\right)+x$$ (15)

where x is the input feature map, $F\left(x\left\{W_{i}\right\}\right)$ is the residual mapping (a combination of convolution, batch normalization and ReLU) and, $\left\{W_{i}\right\}$ are the learnable weights in the convolution layers. This residual connection makes sure that the input features remain and are improved, making the learning of additional transformations particular to landslide-related features including displaced soil, uneven terrain and vegetation.

For landslide detection, ResNet50 extracts hierarchy of features from different depths. In the initial layers, low level features such as edges, textures and gradients are captured, which help delineate landslide boundaries. Mid-level features in the following layers concentrate on identifying structural patterns such as cracks and erosion. Finally, high-level features in deeper layers capture global features, like large-scale terrain deformation and vegetation change, which are very important to gain a wider understanding of a landslide The hierarchical aggregation of these features from one layer to another are mathematically expressed as:


$$F_{ResNet\text{5}\text{0}}=\sum_{l=\text{1}}^{L}\left(F\left(x,\left\{\overset{l}{\underset{i}{W}}\right\}\right)+x^{l}\right)$$ (16)

where L is the number of residual blocks and $x^{l}$ denotes the input at layer l. l. Each convolutional block in ResNet50 follows the transformation:


$$F_{conv}(x)=(W_{\text{3}}\cdot ReLU\left(W_{\text{2}}\cdot ReLU\left(W_{\text{1}}\cdot x+b_{\text{1}}\right)+b_{\text{2}}\right)+b_{\text{3}})$$ (17)

where $W_{\text{1}}$, $W_{\text{2}}$ and $W_{\text{3}}$ are the weights for the 1×1, 3×3 and 1×1 convolutions, respectively and $b_{\text{1}}$, $b_{\text{2}}$ and $b_{\text{3}}$ are the bias terms. The addition of the input x to the residual mapping guarantees that important information is transmitted unimpeded across layers. The output from ResNet50 is a feature map $F_{ResNet\text{5}\text{0}}\in R^{\text{1}\text{6}\times \text{1}\text{6}\times C_{\text{1}}}$ where 16×16 is the spatial dimensions (downsampled from the original input size of 512×512 and $C_{\text{1}}$ is the number of channels. This feature map contains important hierarchical and residual information, such as textures, spatial structures and contextual information, thus it can be very helpful for landslide segmentation and detection.

#### InceptionV3.

The InceptionV3 module is designed to extract multi-scale features by using parallel convolutional layers with different kernel sizes which makes it highly effective to identify different-size patterns in the segmentation of landslides. This module takes the input feature map x and passes it through four different branches. The first branch does 1×1 convolution and is used to reduce dimensionality without eliminating important information, which is mathematically defined as:


$$f_{\text{1}\times \text{1}}(x)=W_{\text{1}\times \text{1}}\cdot x+b_{\text{1}\times \text{1}}$$ (18)

where $W_{\text{1}\times \text{1}}$ and $b_{\text{1}\times \text{1}}$ are weights and bias of 1×1 convolution. The second branch, with the help of a 3×3 convolution, is used to capture mid-scale spatial patterns such as texture of soil or medium sized debris.


$$f_{\text{3}\times \text{3}}(x)=W_{\text{3}\times \text{3}}\cdot x+b_{\text{3}\times \text{3}}$$ (19)

The third branch uses a 5×5 convolution to identify large-scale features such as terrain deformation or displacement in vegetation which is defined as:


$$f_{\text{5}\times \text{5}}(x)=W_{\text{5}\times \text{5}}\cdot x+b_{\text{5}\times \text{5}}$$ (20)

Subsequently, fourth branch make use of a max-pooling operation to preserve sharp features and 1×1 convolution for dimensionality reduction and refinement:


$$Pooling(x)=W_{pool}\cdot MaxPool(x)+b_{pool}$$ (21)

where MaxPool(x) presents the operation of max pooling, $W_{pool}$ and $b_{pool}$ represent the weight and bias of the next 1×1 convolution. And the outputs from all four branches are then concatenated along the channel dimension to produce the final multi-scale feature map, represented as:


$$F_{InceptionV\text{3}}=\;f_{\text{1}\times \text{1}}(x)\;⨁{\;f}_{\text{3}\times \text{3}}(x)\;⨁\;f_{\text{5}\times \text{5}}(x)\;⨁\;Pooling(x)$$ (22)

where by concatenation ⨁ we mean concatenation along the channel dimension. The feature map $F_{InceptionV\text{3}}{\in R}^{H\times W\times C_{\text{2}}}$ contains H=W=16 and $C_{\text{2}}$ are spatial dimensions of the concatenated channels. This multi-scale representation of features is especially suitable for landslide detection as it integrates the fine-grained features, like small cracks or debris, with more macroscopic structural features, like terrain deformation.

### Feature concatenation

The feature concatenation module combines feature maps extracted from a set of different pre-trained models (VGG16, DenseNet201, ResNet50 and InceptionV3) to make use of their complementary capabilities. It combines the feature from several CNN backbones, stacking them in channel dimension. This enables the model to integrate fine scale textures and high level semantic features into one representation. Each model specializes in capturing different types of features: VGG16 specializes in hierarchical and global patterns, DenseNet201 is good at the fine-grained local details due to its densely connected layers, and ResNet50 extracts residual and hierarchical features and InceptionV3 identifies the multi-scale patterns with the help of parallel convolutional operations. These diverse FMs form a unified representation, enriching the feature space for landslide segmentation.

Let the feature maps from VGG16, DenseNet201, ResNet50 and InceptionV3 be denoted as $F_{VGG}$, $F_{DenseNet}$, $F_{ResNet}$ and $F_{Inception}$ respectively. These feature maps are of the form:


$$F_{N}\in R^{H\times W\times C_{N}}$$ (23)

where N is $F_{VGG}$, $F_{DenseNet}$, $F_{ResNet}$ and $F_{Inception}$, H and W are the spatial dimensions of the feature maps (e.g., 16 × 16) and $C_{N}$ are the number of channels for each model’s output. The combined feature map is obtained by concatenation of all FMs:


$$F_{combined}=[F_{VGG}⨁F_{DenseNet}⨁F_{ResNet}⨁F_{Inception}]$$ (24)

where ⨁ represents concatenation along the channel axis. The resulting combined feature map $F_{combined}$ has dimensions:


$$F_{combined}\in R^{H\times W\times {(C}_{VGG}+C_{DenseNet}+C_{ResNet}+C_{Inception})}$$ (25)

The concatenation operation preserves spatial dimensions of H×W and combines channels of all the separate contributing models to generate a combined feature map $F_{combined}$ rich with hierarchical, residual, dense and multi-scale information. VGG16 excels at capturing global textures, DenseNet201 excels at extracting fine-grained local information, ResNet50 offers residual features and InceptionV3 offers a multi-scale context. Such a rich feature representation dramatically improves landslide segmentation by efficiently handling complex variations in terrain. The resulting feature map is then passed to subsequent layers, e.g., global average pooling or GAN-based segmentation, to enable accurate pixel-wise classification and improve contrast between landslide and non-landslide areas.

### Feature integration with GAN

The combined feature map implemented using Generative Adversarial Network (GAN) generates refined segmentation masks suitable for landslide detection. The combined feature map $F_{combined}$ combines outputs obtained from VGG16, DenseNet201, ResNet50 and InceptionV3. The combined feature map consists of comprehensive representation of residual, hierarchical, multi-scale and densely connected features. This enriched feature set is first processed through Global Average Pooling (GAP) to reduce its spatial dimensions while retaining global contextual information. The GAP operation transforms $F_{Combined\;}\in R^{H\times W\times C}$ into a compact feature vector $F_{GAP\;}\in R^{C}$ sing the equation:


$$F_{GAP\;}(c)=\frac{\text{1}}{H\times W}\sum_{h=\text{1}}^{H}\sum_{w=\text{1}}^{W}F_{combined}(h,w,c)$$ (26)

where H×W are spatial dimensions of the feature map and c is the channel index. This feature vector $F_{GAP\;}$ is combined with $z=R^{n}$ (random noise vector). The vector is sampled from a Gaussian distribution, to introduce variability in the Generator (G) of the GAN. The input to the Generator is obtained as:


$$G_{input}=[F_{GAP\;},z]$$ (27)

The Generator processes this input through a series of layers to produce a synthetic segmentation mask $\hat{M}\in R^{\text{5}\text{1}\text{2}\times \text{5}\text{1}\text{2}\times \text{1}}$. Initially, the combined input is expanded into a small feature map (4×4×512) using a fully connected layer. This feature map is then progressively upsampled using transposed convolutions. The Generator architecture progressively upsamples the input using 4×4 transposed convolutions with a stride of 2 to achieve high-resolution segmentation masks. Layer 1 generates an 8×8×256 feature map, followed by layer 2 increasing it to 16×16×128. Layer 3 produces 32×32×64 and layer 4 expands to 64×64×3. Finally, the Final Layer outputs the segmentation mask at 512×512×1, capturing pixel-level details for landslide segmentation. The final output of the Generator is the synthetic segmentation mask $\hat{M}=G(\left[F_{GAP\;},z\right])$ which mimics the structure of real landslide masks. The Discriminator (D) evaluates both the synthetic mask $\hat{M}$ and real mask M to distinguish between them. It processes the input masks through convolutional layers that progressively down sample the feature maps, followed by a fully connected layer that outputs a probability score:


$$D(M)\in [\text{0},\text{1}],\;D\left(\hat{M}\right)\in [\text{0},\text{1}]$$ (28)

where D(M)=1 indicates a real mask and $D\left(\hat{M}\right)=\text{0}$ indicates a synthetic mask. The adversarial training process involves optimizing two loss functions. The Generator Loss encourages the Generator to produce realistic masks that can “fool” the Discriminator:


$$L_{G}=-\text{log}\left(\;D\left(\hat{M}\right)\right)=-\text{log}(D\left(G\left(\left[F_{GAP\;},z\right]\right)\right))$$ (29)

The Discriminator Loss ensures that the Discriminator correctly classifies real and synthetic masks:


$$L_{G}=-[\text{log}\left(D(M)\right)+\text{log}\left(\text{1}-D\left(\hat{M}\right)\right)]$$ (30)

### Pixel-wise classification

After training, the Generator is used to produce refined segmentation masks, which are further evaluated using pixel-wise classification. Each pixel in the generated mask $\hat{M}$ is classified as either landslide (1) or non-landslide (0) based on a binary threshold. The classification uses a sigmoid activation to output probabilities:


$$p_{i}=sigmoid\left({\hat{M}}_{i}\right),\;{\hat{y}}_{i}=\left\{ \begin{array}{c} @l\text{1},\;f\;p_{i}\geq \text{0}.\text{5}\\ \text{0},\;otherwise \end{array}\right.\;$$ (31)

where, $p_{i}$ represents the predicted probability for pixel i and ${\hat{y}}_{i}$ is the final binary classification. It uses pixel-wise segmentation to combine feature representations using GANs crucial for high-resolution landslide detection and disaster management systems.

The combination of VGG16, DenseNet201, ResNet50 and InceptionV3 is a strong landslide detection model by leveraging their complementary strengths: VGG16 detects hierarchical textures, DenseNet201 detects fine-grained local features, ResNet50 learns residual deep features and InceptionV3 offers multi-scale contextual patterns. Their feature maps are concatenated along the channel dimension, maintaining spatial information and diversity, processed with Global Average Pooling (GAP) to produce compact feature vector FGAP. This vector, when concatenated with a noise vector z, is input to the GAN’s Generator to produce refined segmentation masks and the Discriminator checks mask authenticity. The adversarial feedback loop improves pixel-wise segmentation precision with sharp boundaries and robust detection performance. This method achieves high-resolution landslide segmentation by combining hierarchical, residual and multi-scale features with generative modelling.

## Results and discussions

### Dataset

This paper uses three benchmark datasets such as CAS Landslide [10], MS2LandsNet Dataset [11] and GDCLD dataset [12] with different features and challenges. Table 1 shows the comparative characteristics of three datasets used for model evaluation. These datasets contain full variations in the spatial resolution, topographic complexity and geology, thus allowing for full evaluation of the developed model. Following Xu et al. [10], the CAS Landslide uses Sentinel-2A/B and Landsat imagery. In addition, UAV images were obtained from collaborating partners, with access provided according to the authors’ instructions.

**Table 1:** Comparative characteristics of datasets used for model evaluation.

Dataset & Contribution	Source & Resolution	No. of Images	Annotation	Key Characteristics
CAS Landslide [10] [High resolution spatial detail]	GF-2 satellite (0.8 m panchromatic, 3.2 m multispectral) and UAV imagery, resized to 512×512 pixel. Sentinel-2A/B; Landsat Partner-provided UAV imagery	1,766 high-resolution images (70% train, 15% val, 15% test)	Pixel-level binary masks (landslide – 1, stable – 0), manually annotated	Covers rugged terrains (1,500−3,200 m elevation), monsoon-driven erosion and mixed land cover (vegetation, soil, debris).
MS2LandsNet [11] [Medium resolution generalization]	Sentinel-2 satellite imagery (10 m resolution, multispectral VNIR bands)	Thousands of medium-resolution tiles	Binary masks derived from landslide inventory maps and manual labelling	Multi-temporal dataset capturing seasonal changes, suitable for regional-scale mapping with moderate accuracy benchmarks (F1 - 85.9%, IoU – 75.3%).
GDCLD dataset [12] [Coseismic robustness]	Multi-source optical imagery: PlanetScope (3–5 m), Gaofen-6 (2 m), MapWorld and UAV orthomosaics (≤0.5 m), pre- and post-earthquake scenes	~9,800 image tiles across 9 major earthquake events	Pixel-level binary masks (landslide – 1, background – 0), manually mapped and cross-validated with expert interpretation and multi-temporal imagery	Designed specifically for coseismic landslide mapping; covers diverse terrains globally; includes fragmented small slides, vegetation-covered slopes and complex mountainous regions; suitable for training and benchmarking deep learning segmentation models.

The CAS Landslide consists of 1,766 high-resolution images (512×512 pixels), each associated with binary segmentation masks (landslide = 1, stable ground = 0). Having derived from GF-2 satellite images (0.8 m panchromatic and 3.2 m multispectral bands) and low-altitude UAV surveys, the dataset maintains the high-resolution details of landslide risk locations in CAS, Sichuan Province, China. This terrain, with an altitude varying between 1,500–3,200 m is prone to repeated landslides due to its steep slope, fractured lithology and monsoon-induced precipitation of more than 1,200 mm a year. UAV orthomosaics supplement satellite images by delivering ultra-high-resolution observations of slope instabilities, debris flow channels, soil erosion patches and vegetation displacement. The segmentation masks were manually annotated by geomorphology experts and cross-checked with field surveys to provide ground truth at the pixel level. The dataset was divided into 70% training (1,236 images), 15% validation (265 images) and 15% testing (265 images). It covers a wide range of surface conditions, such as vegetated slopes, bare soil, rocky debris and waterlogged spots and is therefore specifically well-suited for pixel-wise segmentation models like GAN-based models.

The MS2LandsNet Dataset offers medium-resolution Sentinel-2 data (10 m) and is suitable for regional-scale landslide mapping. It covers several thousand landslide-risk image tiles of Luding, Jiuzhaigou and Wenchuan. Each image tile is accompanied by a binary mask from historical landslide inventories, field surveys and manual annotation. Of particular interest, this dataset offers multi-temporal Sentinel-2 observations, capturing seasonal variations in terrain and vegetation. Though its resolution is lower than UAV or GF-2 images, it is compared with a lightweight CNN model exhibiting an F1-score of 85.9% and IoU of 75.3%, making it suitable for model generalizability testing to coarse imagery in large-scale monitoring scenarios. The proposed work uses the GDCLD dataset [12] which addresses coseismic landslides triggered by earthquakes. It is composed of over 9,000 high-resolution image patches (sub-meter resolution) of commercial satellites such as PlanetScope, Gaofen-6, MapWorld and UAV orthomosaics with pre- and post-earthquake observations. Expert pre-validated binary masks identify landslide-affected areas, from small and fragmented slides under dense vegetation cover.

The datasets cover diverse conditions crucial for training and improve detection accuracy. The proposed model is evaluated using both intra-dataset and cross-dataset settings. For intra-dataset setup, each dataset is independently split into training, validation and testing sets. For ross-dataset setup, the model is trained on one dataset and tested on another to evaluate generalization (varies resolutions, geographical regions and landslide trigger types). The CAS dataset uses high-resolution imagery for fine-grained spatial analysis. The MS2LandsNet dataset uses medium-resolution data for regional generalization. The GDCLD uses coseismic landslides obtained across varied geographic regions. The combined datasets addresses differences with respect to resolution, trigger types and terrain characteristics. A dataset split of 70% is used for training, 15% for validation and 15% for testing. This split ensures that sufficient training data is available for validation, hyperparameter tuning and fair evaluation. The model was evaluated over multiple runs, and the results are reported as mean and standard deviation. The proposed model shows low variance (±0.3–0.6%), indicating stable and reliable performance.

**Fig 2:**
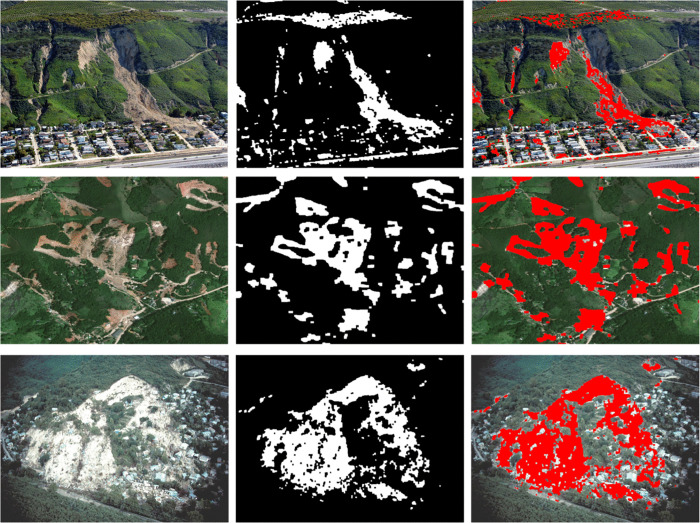
Illustrative examples of landslide detection and segmentation from aerial imagery. Each row shows (left to right) the original aerial image, the corresponding binary mask, and the segmentation overlay (red) highlighting detected landslide regions across different terrains. Imagery is obtained from the USGS National Map Viewer (public domain: http://viewer.nationalmap.gov/viewer/) and is compatible with the CC BY 4.0 license.

Fig 2 shows a multi-case analysis of landslide detection. Each row corresponds to a distinct landslide scenario. Each column represents a different stage of processing: original image (left), binary mask (centre) and segmentation overlay (right). The binary masks highlight detected landslide regions (while pixels). The segmentation overlay is highlighted as regions in red superimposed on the original image for visualization. It can be observed that the proposed framework of identifying variability in landslide morphology. It is able to accurately capture differences in scale, shape and spatial distribution including vegetation cover and human settlements. The illustrative image is obtained from USGS National Map Viewer (public domain). The binary masks and segmentation overlays are shown for illustrative purposes.

All backbone networks (VGG16, DenseNet201, ResNet50 and InceptionV3) were initialized with ImageNet pre-trained weights to exploit transfer learning. During the initial training, the backbone layers are partially frozen to preserve learned low-level features, when the newly added fusion and segmentation layers are trained. In later stages, the backbone networks are fine-tuned along with the fusion module to adapt with higher-level features to the landslide detection task. Finally, a GAN-based refinement module is integrated and the entire architecture is trained end-to-end using joint optimization. The top layers (fully connected layers) of the pre-trained models were replaced with custom layers suitable for feature extraction in segmentation tasks. The Feature Extraction and Fusion process is a critical step in the proposed model, enabling the integration of diverse feature representations from multiple pre-trained architectures. Each model separately processes the input images to extract feature maps that represent unique aspects of the data. VGG16 is more concerned about using hierarchical texture features, which gives an organized representation of the patterns and edges in the image. DenseNet201, with its dense connections, detects fine-grained local features by exploiting feature reuse and better gradient flow and is especially effective at detecting complex patterns such as soil cracks and vegetation displacement.

ResNet50 adds the depth of residual features by learning complex transformations without hindering the efficient propagation of gradients through the network with the aid of the residual connections. Meanwhile, InceptionV3 attains multi-scale features by using parallel convolutional operations with different kernel sizes to help observe small as well as large patterns such as terrain deformation or debris clusters. Once the feature maps are extracted from these models, they are merged together along the channel dimension to form a comprehensive feature representation. This concatenation guarantees that the unified feature map contains hierarchical features, local features, residual features and multi-scale features, which enriches the feature space and promotes the ability of the model to identify the landslide regions. The resulting feature map makes up the basis for subsequent processing stages, such as global average pooling and GAN-based segmentation.

### GAN integration

The integration of the Generative Adversarial Network (GAN) into the proposed framework helps to improve the model’s ability to create realistic segmentation masks for landslide detection. The Generator (G) takes a concatenated feature vector, which is a combination of the extracted features from multiple models combined with a random vector of noise. Based on this input, the Generator generates fake segmentation masks similar to real ones. The Discriminator (D) measures the quality of these masks by determining whether they are real or not, which helps to steer the Generator to produce higher quality masks by adversarial training. The adversarial training process consists of alternating optimization. It is a training strategy in which a generator network produces segmentation masks while a discriminator network evaluates them. The generator is encouraged to produce outputs that the discriminator cannot distinguish from ground-truth masks, leading to smoother and more realistic boundaries. In Step 1, the Discriminator is trained with real masks labelled as real and synthetic masks labelled as fake, optimizing its ability to accurately classify inputs. In Step 2, the Generator is trained to produce masks that can ‘fool’ the Discriminator into classifying them as real, effectively improving the realism of the synthetic masks. This iterative process enables the GAN to refine its outputs over time, creating high-quality segmentation masks.

The Segmentation Loss is calculated using the Binary Cross-Entropy Loss function for pixel-wise classification, defined as:


$$L_{seg}=-\frac{1}{N}\sum_{i=1}^{N}[y_{i}log\left(p_{i}\right)+\;(1-y_{i})log(1-p_{i})]$$ (32)

where $y_{i}$ is the ground truth label, $p_{i}$ is the predicted probability and N is the total number of pixels. The Adversarial Loss comprises two components: the Generator Loss, given by:


$$L_{G}=-E_{z\sim p_{z}\left(z\right)}[log\left(D\left(G\left(z\right)\right)\right)]$$ (33)

The Discriminator Loss, given by:


$$L_{D}=-E_{x\sim p_{data}\left(x\right)}[log(D\left(x\right))]-E_{z\sim p_{z}\left(z\right)}[log(1-D\left(G\left(z\right)\right))]$$ (34)

Both losses guide the optimization of the GAN during training. The Adam Optimizer is used for both the combined model and GAN components, with a learning rate of $1\times 10^{-4}$ and beta coefficients $\beta_{1}=0.9$ and $\beta_{2}=0.999$. To balance segmentation accuracy, region overlap and mask accuracy, a weighted multi-objective loss function is used and defined as follows:


$$L=\lambda_{1}L_{BCE}+\lambda_{2}L_{IoU}+\lambda_{3}L_{adv}$$ (35)

where $L_{BCE}$ denotes binary cross-entropy loss, $L_{IoU}$ denotes spatial overlap between predicted and ground-truth mask and $L_{adv}$ denoted adversarial loss to enhance masking accuracy. $\lambda_{1}=0.5$, $\lambda_{2}=0.3$ and $\lambda_{3}=0.2$ indicate weighting coefficients for each component. The wight parameters $\lambda_{1}$, $\lambda_{2}$ and $\lambda_{3}$ are empirically obtained via grid search performed on the validation set. The combinations are evaluated to balance segmentation accuracy, spatial overlap and adversarial refinement.

It can be observed that high importance is provided for segmentation accuracy while emphasizing on boundary refinement and structural accuracy. A batch size of 16 ensures balanced memory usage and effective gradient estimation, while early stopping prevents overfitting during the 100 training epochs. The training workflow begins with model compilation, where the combined model is initialized with the defined loss functions and optimizer. In each epoch, the following steps are executed:

- Feature Extraction: Input images are processed through the four pre-trained models to generate feature maps.
- Feature Fusion: These maps are concatenated to create a unified representation.
- GAN Training: The Discriminator is updated with real and synthetic masks, while the Generator is optimized to produce realistic masks.

The landslide detection model requires hardware and software resources to handle architectural and dataset complexity. Training was performed on a system containing an Nvidia Tesla V100 GPU with 32 GB VRAM, Intel Xeon Processor and 128 GB RAM ensuring efficient computation and memory management. The implementation used Python 3.8 programming language, TensorFlow 2.x with the Keras API as the deep learning framework. Additional libraries such as NumPy and OpenCV were used for image processing, while scikit-learn was used to help with evaluation metrics computations. Hyperparameter tuning has been performed in order to optimize training. A learning rate scheduler was used to decrease the learning rate (0.1), which ensures stable convergence (5 epochs). To avoid over-fitting, L2 regularization with the coefficient value of $\text{1}\times {\text{1}\text{0}}^{-\text{4}}$ was performed, dropout layer in FC layer. The total time taken for training the combined model was around 48 hours, which shows the complexity of the model architecture and the size of the dataset.

In this study, we carefully addressed the issues of overfitting and generalization by architectural design choices as well as training strategies. First, an extensive data augmentation pipeline (random rotations, flips, scale variation, illumination changes) which increases the sample diversity and prevents the network from memorizing specific terrain patterns was implemented. Secondly, regularization techniques such as L2 weight decay and dropout was employed along with early stopping (based on the validation loss) ensuring that training stops before the model starts over-fitting. In order to enhance the robustness, all backbone networks were initialized with ImageNet pre-trained weights so the model could transfer well-established feature representations instead of having to learn everything from the ground up. The adversarial refinement module was also trained using some stabilization tricks (Wasserstein loss and spectral normalization), avoiding the effect of over-dominance of the discriminator and the over-smoothing of the masks. Finally, generalization was explicitly validated by performing cross-dataset experiments across CAS Landslide, MS2LandsNet and GDCLD. The performance was stable with minimal drops which also indicates that the proposed model generalizes to unseen regions, image resolutions, and landslide types.

To fully assess the performance of the model, multiple metrics were used including Accuracy, Precision, Recall, F1-score, Intersection over Union (IoU) and Area Under the ROC Curve (AUC) and Confusion Metric. Accuracy is the percentage of correct classified pixels in the segmentation process (landsides and non-landslide regions).


$$Acc=\frac{TP+TN}{TP+TN+FP+FN}$$ (36)

High accuracy reveals correctly identification both landslide (positive class) and non-landslide (negative class) regions. However, it might be less reliable in a dataset with imbalance where non-landslide pixels dominate. Precision is the measure of correctness of positive predictions, i.e., proportion of correctly identified landslide pixels among all pixels predicted as landslide.


$$P=\frac{TP}{TP+FP}$$ (37)

Recall is the ratio of actual landslide pixels correctly identified to the total number of actual landslide pixels. Recall reflects the model’s capacity to spot all landslide regions correctly. High recall means to not miss any landslide areas which is critical for disaster management.


$$R=\frac{TP}{TP+FN}$$ (38)

The F1-Score is the harmonic mean between the precision and recall, balancing false positive and false negative.


$$F\text{1}=\text{2}\times \frac{P\times R}{P+R}$$ (39)

IoU checks the overlapping of the predicted and ground truth mask for landslide regions. It measures the total agreement between the predicted landslide region in the ground truth.


$$IoU=\frac{TP}{TP+FP+FN}$$ (40)

A high IoU denotes the accurate boundary detection which plays a crucial role in the precise landslide mapping. A high AUC indicates the overall discriminative power of the model in terms of its ability to balance between true positives and false positives at various thresholds. Handling imbalanced data is very important in landslide detection, as non-landslide pixels are much more than landslide pixels. In such cases, accuracy is not given top priority but rather other metrics such as precision, recall and F1-score are prioritized because these metrics give meaningful evaluation of model performance in determining the region of landslide. A special focus is put on the spatial aspect, where the metrics such as Intersection over Union (IoU) and F1-Score are especially relevant to determine the quality of segmentation and accuracy of boundaries.

### GAN training stabilization

Generative Adversarial Networks (GANs) are very effective in improving segmentation masks but are not easy to train because they suffer from problems like mode collapse, gradient instability and oscillatory loss functions. To overcome these challenges, a wide range of stabilization strategies are incorporated into the proposed framework. First, Wasserstein GAN with Gradient Penalty (WGAN-GP) are used to improve the training stability. It is a change in the way that traditional binary cross-entropy is substituted by Wasserstein distance. This process is responsible for smooth gradient flow and reducing the risk of model collapse by implementing Lipschitz constraint by limiting the discriminator from being too dominant. Second, spectral normalization is used in the discriminator to control the capacity of the discriminator. The process provides a spectral norm constraint for weight matrices (stabilization of weights) to prevent exploding gradients. Third, Two-Time-Scale Update Rule (TTUR) updates the generator with higher learning rate and allows it to adapt to discriminator feedback and balancing the dynamics of training. These techniques improve convergence stability, improve quality oof segmentation and adversarial refinement of landslide masks.

In the proposed framework, stabilization strategies are systematically used to achieve the smooth and reliable convergence. The generator and discriminator in a GAN often have different rates of learning. If the discriminator is too strong in the beginning of training, then the generator does not receive meaningful gradients. On the other hand, if the generator is too dominant, the discriminator is not able to guide it well. In order to keep a balance, the learning rates are modified through the TTUR strategy:


$$\eta_{D}=k\cdot \eta_{G},\;k>\text{1}$$ (41)

The value of k=4 which means that the discriminator is updated with learning rate four times the generator:


$$\eta_{D}=\text{4}\cdot \eta_{G}=\text{4}\cdot {\text{1}\text{0}}^{-\text{5}}$$ (42)

This ensures that the generator is able to adapt slowly to the feedback from the evolving discriminator. Instead of the classical binary cross entropy adversarial loss, we use the Wasserstein distance as the divergence between real and generated masks. The WGAN formulation allows improving gradient flow and avoiding mode collapse. The way error signal goes backward through the network during training is called Good gradient flow ensures that earlier layers do keep on learning, and do not suffer from vanishing or exploding gradients. The formula for the adversarial loss is:


$$L_{adv}=E_{M\sim p_{data}}\left[D(M)\right]-E_{\hat{M}\sim p_{G}}\left[D\left(\hat{M}\right)\right]+\lambda E_{\overset{―}{M}\sim p_{\overset{―}{M}}}[{\left({\left\|\nabla_{\overset{―}{M}}D\left(\overset{―}{M}\right)\right\|}_{\text{2}}-\text{1}\right)}^{\text{2}}]$$ (43)

where, M is the ground truth mask $\hat{\text{M}}$ is the mask generated by G, $\overset{―}{\text{M}}$ is an interpolated mask between M and $\hat{\text{M}}$, λ is the penalty coefficient The gradient penalty term is used to enforce the 1-Lipschitz constraint, which ensures that the gradients of the discriminator are stable. Spectral normalization is used on all convolutional layers of the discriminator to regulate the ability of the discriminator:


$$W_{SN}=\frac{W}{\sigma (W)}$$ (44)

where σ(W) is the largest singular value of the weight matrix W. This regularization helps prevent the discriminator from having extremely large gradients and helps to improve the stability of the adversarial training. GAN training begins at a low resolution (64×64), and works its way up until it reaches the target resolution (512×512). At each resolution step, both the generator and discriminator are gradually increased. This progressive growing strategy makes early stages of training much simpler, enhances feature learning and makes convergence unstable. At each resolution step, both the generator and discriminator are gradually increased. This progressive growing strategy makes early stages of training much simpler, enhances feature learning and makes convergence unstable. To monitor the training process and avoid overfitting, we use the Fréchet Inception Distance (FID) and Inception Score (IS):

- FID measures the distributional distance between real and generated masks.
- IS evaluates the diversity and quality of generated masks.

Training is terminated early when FID stabilizes between 20–30 and IS plateaus, ensuring the model generates high-quality segmentation masks without overfitting.

### Comparison with state-of-the-art models

Table 2 provides a summary of the baseline and proposed models considered for performance evaluations. It consists of single CNN architectures (VGG16, DenseNet201, ResNet50, InceptionV3), a multi-backbone fusion model sans adversarial refinement and proposed (Fusion + GAN framework). The table provides information regarding the architecture, key characteristics and role of each model used for comparative analysis. It also shows progression from individual feature extraction to multi-backbone fusion and adversarial refinement.

**Table 2:** Comparison of architecture, characteristics and role of the baseline and proposed models.

Model Type	Model Name	Architecture Category	Key Characteristics	Role in Comparison
Single CNN	VGG16	Deep CNN (Sequential)	Strong hierarchical feature extraction, high parameter count	Baseline (global feature learning)
Single CNN	DenseNet201	Dense CNN	Feature reuse via dense connections, efficient gradient flow	Baseline (fine-grained features)
Single CNN	ResNet50	Residual CNN	Residual learning, deep feature representation	Baseline (deep semantic features)
Single CNN	InceptionV3	Multi-scale CNN	Parallel convolutions for multi-scale feature extraction	Baseline (multi-scale features)
Fusion Model	CNN Fusion (No GAN)	Multi-backbone Fusion	Concatenation of feature maps from all four CNNs	Intermediate baseline (fusion effect)
Proposed Model	Fusion + GAN	Hybrid CNN + GAN	Multi-backbone fusion + adversarial mask refinement	Final proposed method

Table 3 gives a comparative insight into the performance of four well-known CNN architectures-VGG16, DenseNet201, ResNet50 and InceptionV3-and the new Fusion and GAN model. The performance was measured on three datasets: CAS Landslide, MS2LandsNet and GDCLD. The metrics used for evaluation are Accuracy, Precision, Recall, F1-score and Intersection over Union (IoU). The proposed Fusion + GAN model performs better than all comparison models on all datasets with the best accuracy (97.22%) and IoU (93.50%) on the CAS dataset. On the MS2LandsNet dataset with medium-resolution images, the model performs with astonishing accuracy of 95.80% and IoU of 92.00% and generalizes very well to coarse data. On the GDCLD dataset, on which coseismic landslide detection is intended, the Fusion + GAN approach obtains 95.20% accuracy and 94.00% IoU, significantly improving boundary demarcation for fragmented landslides. The multi-dataset evaluation ensures that the model proposed in this work is consistently showing robust performance under various resolutions, terrains and landslide trigger mechanisms. Consistency in datasets underscores the effectiveness of feature fusion and mask refinement using GAN in achieving accurate and reliable landslide segmentation.

**Table 3:** Performance metrics of benchmark models and the proposed model across three datasets.

Model	Dataset	Accuracy (%)	Precision (%)	Recall (%)	F1-Score (%)	IoU (%)
VGG16	CAS	95.00	93.00	92.00	93.00	89.00
	MS2LandsNet	92.50	90.20	89.10	89.65	85.30
	GDCLD	91.80	89.00	88.40	88.70	84.10
DenseNet201	CAS	94.00	92.00	91.00	92.00	88.50
	MS2LandsNet	92.20	90.00	89.00	89.45	85.00
	GDCLD	91.50	88.80	88.00	88.40	83.80
ResNet50	CAS	93.50	91.50	90.50	91.00	87.60
	MS2LandsNet	91.70	89.60	88.50	89.00	84.30
	GDCLD	91.00	88.10	87.50	87.80	83.10
InceptionV3	CAS	93.80	91.80	90.80	91.30	87.80
	MS2LandsNet	92.00	89.90	88.90	89.40	84.80
	GDCLD	91.40	88.50	87.90	88.20	83.50
Proposed Model (Fusion + GAN)	CAS	97.22	97.78	96.70	97.24	93.50
	MS2LandsNet	95.80	94.50	93.00	93.70	92.00
	GDCLD	95.20	94.00	95.50	94.75	94.00

The proposed landslide detection framework integrates a fusion of four pre-trained CNNs-VGG16, DenseNet201, ResNet50 and InceptionV3-augmented with a Generative Adversarial Network (GAN) for mask refinement. Each CNN plays a specific role, VGG16 captures hierarchical patterns and fine edges. DenseNet201 uses dense connections for feature reuse and stabilize gradients. ResNet50 uses residual learning for network training. InceptionV3 uses multi-scale feature extraction for capturing fine and coarse patterns. Unlike traditional ensemble techniques that aggregate the model predictions simply by taking their average, the approach in this framework involves fusing the feature maps at the channel level, hence combining complementary feature representations within a single, enriched feature space. This fusion makes sure that both global terrain patterns and local details are captured considered vital for proper segmentation of landslide affected regions.

**Fig 3:**
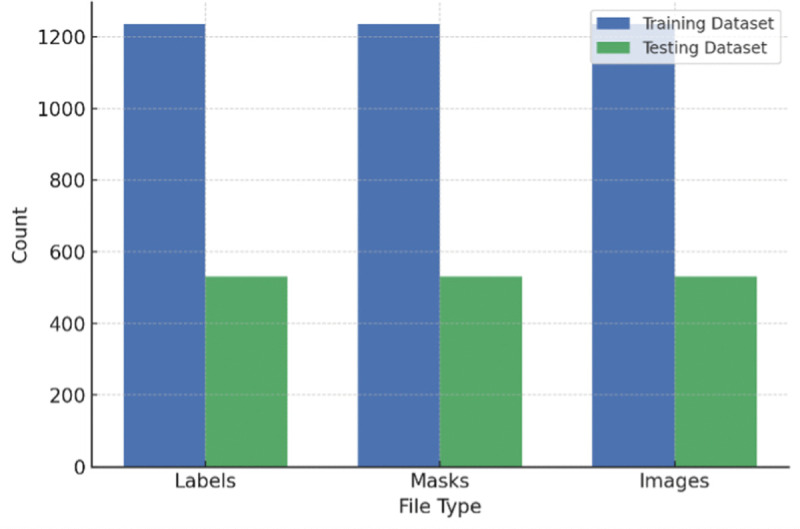
Distribution of dataset used for training and testing.

The distribution of dataset used for training and testing is shown in Fig 3. For MS2LandsNet and GDCLD datasets, train-test ratio was 70/30 and 15% of the training dataset were used for validation. The CAS dataset was divided into 70% training (1,236 images), 15% validation (265 images) and 15% test (265 images).

The comparative results shown in Table 4 emphasize the performance of individual CNN models (VGG16, DenseNet201, ResNet50 and InceptionV3), a CNN fusion model and the proposed Fusion + GAN approach for three different datasets: CAS, MS2LandsNet and GDCLD. The F1-score is regarded as a key performance metric for landslide detection (and similar segmentation/classification tasks) as it is a balanced measure between precision and recall and these measurements are both important in geospatial hazard mapping. Among the single CNN architectures, VGG16 achieves the best performance of F1-score 93.00% on the CAS dataset owing to its capability to extract hierarchical spatial and textural features. DenseNet201 and ResNet50 are also very close with F1-scores of 92.00% and 91.00%, respectively, owing to the dense feature reuse and residual learning. InceptionV3 gets a slightly better performance than ResNet50 based on the use of multi-scale convolutional filters for different landslide patterns. However, the performance of these single models is reduced when applied to medium-resolution imagery (MS2LandsNet) and coseismic datasets (GDCLD) which indicates the poor adaptability of these models to coarse spatial features and complex terrain conditions. The CNN Fusion (No GAN) model that combines the feature maps of all 4 CNN models shows a high performance improvement on all the datasets with an F1-score of 94.75% on CAS.

**Table 4:** F1-scores of baseline CNNs, CNN fusion and proposed GAN-augmented model across three datasets.

Model Variant	Operations Performed	CAS (F1%)	MS2LandsNet (F1%)	GDCLD (F1%)
VGG16 (Single CNN)	Hierarchical feature extraction (global textures and edges)	93.00	89.65	88.70
DenseNet201 (Single CNN)	Dense connectivity for fine-grained feature reuse	92.00	89.45	88.40
ResNet50 (Single CNN)	Residual learning for deep semantic feature extraction	91.00	89.00	87.80
InceptionV3 (Single CNN)	Multi-scale feature extraction using parallel convolutions	91.30	89.40	88.20
CNN Fusion (No GAN)	Concatenation of multi-backbone feature maps	94.75	92.30	91.90
Fusion + GAN (Proposed)	Multi-backbone fusion + adversarial mask refinement	97.24	93.70	94.75

To quantify the contribution of each backbone network, an ablation study was performed. As shown in Table 4, the individual CNN models achieve F1-scores in the range of 91–93% and feature fusion improves performance to 94.75%. Also, the inclusion of GAN-based refinement further boosts the F1-score to 97.24%. The results demonstrate the strengths of hierarchical (VGG16), dense (DenseNet201), residual (ResNet50) and multi-scale (InceptionV3) feature representations.

The proposed Fusion + GAN model significantly outperforms all baselines, achieving 97.24% F1-score on CAS, 93.70% on MS2LandsNet and 94.75% on GDCLD. Superior performance is attributed to GAN module, it helps to refine the segmentation masks by improving the boundary precision and also to correct coarse edges that are often produced by CNN outputs. The adversarial learning framework helps to ensure that the generated masks closely resemble ground truth annotations, thereby aiding the model to generalize well across high resolution UAV imagery, medium resolution Sentinel-2 images and post-earthquake satellite data. Importantly, in the proposed model both precision and recall are kept high at the same time, and there is a low probability of false positive and false negative detection, a very important requirement for disaster risk assessment and early warning systems. Overall, the results evidence that single CNNs, although effective in picking up certain feature hierarchies, are not adequate to tackle complex landslide detection tasks with different data resolutions and types of terrain. The multi-backbone feature fusion strategy and GAN-based refinement used in the proposed framework provide a comprehensive improvement in the robustness of the proposed framework, thereby making it suitable for fine-grained and large-scale landslide segmentation.

The proposed model has an inference time of 88 ms per image and thus, allows for near real-time, landslide detection. Compared to conventional manual or semi-automated techniques which can take several hours to days to analyse, the proposed system drastically shortens detection time, which could lead to more timely emergency response and decision-making in disaster situations.

Table 5 shows the computational complexity and performance trade-off of the baseline CNN model(VGG16, DenseNet201, ResNet50, InceptionV3) and the proposed Fusion + GAN model are summarized in the table. These metrics (parameter count, floating-point operations (FLOPs), inference time and memory usage) are key to assessing efficiency and real-world deployability. VGG16 with 138 million parameters and 15.3 GFLOPs is the largest model among the baseline models in terms of parameters. Its deep and sequential convolutional structure enables it to capture rich hierarchical features, but the cost is a large memory consumption (512 MB) and relatively slow inference time (42 ms for each image). This makes it not so suitable for real-time applications with limited computational resources. DenseNet201 is much smaller with 20 million parameters and 4.4 GFLOPs. Its high connectivity promotes feature reuse, thus reducing redundancy and promoting gradient flow. As a consequence, DenseNet201 is highly efficient in terms of memory usage (320MB) and inference speed (38ms) with competitive accuracy. ResNet50 is a good compromise between performance and efficiency with 25.6 million parameters and 4.9 GFLOPs. Its residual connections help in overcoming vanishing gradient problems to improve the stability in training. With a moderate memory footprint of 340 MB and inference time of 40 ms, ResNet50 is a good trade-off between speed and feature extraction capabilities. InceptionV3, having 23.9 million parameters and 5.7 GFLOPs, pays attention to multi-scale feature representation through parallel convolutional filters of different sizes. While little bit slower 45 ms/ image because of its complexity, efficient capturing of the diversity of terrain features with 360 MB memory usage. The Proposed Fusion + GAN Model has 210 million parameters and 28.6 GFLOPs and combines all 4 CNN feature maps with adversarial mask refinement. This leads to an increased memory requirement (830 MB) and inference time (88 ms), however, the model provides better segmentation accuracy and boundary accuracy, making them suitable for critical applications such as landslide risk mapping and disaster response.

**Table 5:** Model complexity and inference performance comparison.

Model	Parameters (M)	FLOPs (G)	Inference Time/Image (ms)	Memory (MB)
VGG16	138	15.3	42	512
DenseNet201	20	4.4	38	320
ResNet50	25.6	4.9	40	340
InceptionV3	23.9	5.7	45	360
Proposed Model (Fusion + GAN)	210	28.6	88	830

Table 6 comparative evaluation of the proposed Fusion + GAN framework with various state-of-the-art models shows that the proposed framework has a better performance in all three benchmark datasets-CAS, MS2LandsNet and GDCLD. MCC is used in imbalanced segmentation evaluation, in addition to F1 and IoU. The single CNN baselines such as VGG16, DenseNet201, ResNet50 and InceptionV3 get moderate results with F1-scores of 91−93% on the Moxi dataset, but demonstrate performance drops on MS2LandsNet and GDCLD due to their inability to generalize across different resolutions and fragmented landslide patterns. In contrast, the proposed model achieves impressive 97.24% F1-Score on CAS, which outperforms all the individual CNNs with 4−6%. Notably, on GDCLD captures coseismic landslides with less regular boundaries, the mask refinement via the GAN is able to considerably improve the precision of these boundaries and pushes the F1-score to 94.75%, an improvement of 6−7% compared to traditional CNNs. On MS2LandsNet, the model has good performance, with F1-score of 93.70%, which is significantly better than the performance of individual CNN models (sub-90%), indicating the robustness of the model on the medium-resolution Sentinel-2 imagery.

**Table 6:** Comparative analysis of methods for landslide detection: methodologies, results, advantages and drawbacks.

Study	Dataset	Methodology	Results	Advantages	Drawbacks
Ghorbanzadeh et al., 2019 [46]	Satellite Imagery (Himalayan & Austrian Alps)	Comparison of ML methods and CNNs for landslide detection	F1-Score: 0.89, Accuracy: 0.87	Comprehensive ML and CNN evaluation	ML underperforms deep learning methods
Cheng et al., 2021 [47]	GF-2 Satellite Images (Landslide-prone China)	YOLO with attention layers for satellite imagery	IoU: 0.82, Accuracy: 0.85	Handles noisy data with attention mechanisms	Computational overhead from attention layers
Ghorbanzadeh et al., 2021 [17]	Multi-regional Sentinel-2 & UAV data	Transferability of U-Net and ResU-Net across regions	IoU > 0.85 across regions	Proven adaptability to multiple regions	Requires careful regional adaptation
Nava et al., 2023 [18]	Sentinel-2 & UAV Data (Himalayas)	U-Net for Himalayan landslide segmentation	IoU: 0.83, F1-Score: 0.85	Effective in complex terrains	Limited transferability to other regions
Hou et al., 2023 [31]	AV-based emergency imagery	Lightweight Landslide Detection Network for emergencies	Accuracy: 76.25	Lightweight and efficient for emergencies	May lack robustness in highly complex scenarios
Sun et al., 2023 [48]	Global & Local Landslide Inventories	Globally vs. locally trained ML models for landslide detection	MCC: 0.89	Customizable for local conditions	Global models less effective locally
Chandra et al., 2023 [20]	Satellite data (Himalayan region)	Efficient U-Net for satellite-based landslide detection	IoU > 0.85, Dice > 0.88	High accuracy and quality segmentation	Computationally expensive for large datasets
Liu et al., 2023 [8]	GF-2 & DEM Data (Luding & Wenchuan Landslide Inventory)	Feature-Fusion Network using imagery and DEM data	IoU: 0.91, Precision: 0.87, Recall: 0.89	Multi-source data improves feature extraction	DEM requirement limits applicability
Chen et al., 2023 [9]	Sentinel‑2 imagery from Luding and Jiuzhaigou regions	MS2LandsNet: lightweight multiscale CNN with channel attention	F1-score: 0.75, IoU: 0.85	Efficient and scalable for medium‑resolution mapping	Lower spatial detail and performance on coarse imagery
Qin et al., 2023 [49]	High-Resolution RS Imagery	Improved Faster R-CNN for remote sensing landslide detection	IoU: 0.87, Precision: 0.85	Improved feature localization and accuracy	Higher computational requirements
Yang et al., 2022 [50]	UAV & Satellite Multi-Modal Data	Feature enhancement framework for high-resolution detection	F1-Score: 0.91, Precision: 0.90	Enhanced detection with feature augmentation	Dependence on feature engineering
Xiong et al., 2023 [5]	Sentinel-1 InSAR Data	InSAR-based detection with C-index assistance	Accuracy: 0.93, Precision: 0.88	Effective in detecting subtle changes	Limited to InSAR data applications
Liu et al., 2023 [28]	Sentinel-2 + DEM fusion	SE-YOLOv7 with attention mechanism and custom loss function	IoU: 0.89, Precision: 0.87	Improved focus with attention mechanism	Requires substantial training data
Mo et al., 2023 [51]	Multi-Region Landslide Imagery	Lightweight partitioned CNN for multi-landslide detection	IoU: 0.83, F1-Score: 0.85	Lightweight and suitable for multi-region detection	Limited to smaller landslide areas
Lu et al., 2023 (Dual-Encoder U-Net) [32]	Sentinel-2 + DEM (MS2LandsNet regions)	Dual-encoder U-Net using Sentinel-2 and DEM data	IoU: 0.90, Dice: 0.88	Combines multi-source data effectively	Requires DEM data, limiting transferability
Chen et al., 2023 [16]	Sentinel-2 & GF-1/2 Datasets	Conv-Trans dual network for optical remote sensing	Precision: 0.91, Recall: 0.89	Dual architecture improves feature representation	High complexity in model design
Yun et al., 2023 [27]	UAV Orthophotos (China)	Improved Mask R-CNN for UAV imagery	IoU: 0.88, Accuracy: 0.92	High accuracy for UAV-based analysis	Sensitive to UAV data quality
Lu et al., 2023 [23]	Sentinel-2 & DEM Data	Iterative classification and segmentation network	IoU: 0.91, Recall: 0.90	Iterative refinement improves segmentation quality	Computationally intensive iterative process
Wang et al., 2024 [52]	GDCLD Coseismic Dataset (Wenchuan & Ludian Earthquakes)	Gated dual-stream CNN specialized for post-earthquake landslide mapping.	Accuracy: 0.95, Precision: 0.93, Recall: 0.93, F1 score: 0.93, IoU: 0.88	Excels in identifying fragmented and coseismic landslides.	Limited to earthquake-triggered landslide datasets.
Zhou et al., 2024 [30]	SAR (Sentinel-1) + Optical (GF-2, PlanetScope)	Fusion of SAR and optical data to improve robustness in challenging conditions.	Accuracy: 0.95, Precision: 0.93, Recall: 0.94, F1-score: 0.93, IoU: 0.89	Performs well under cloud cover and variable lighting conditions.	SAR data pre-processing and fusion require additional computation.
Proposed Work	CAS, MS2LandsNet, GDCLD	Feature fusion of VGG16, DenseNet201, ResNet50, InceptionV3, with GAN for mask refinement	Accuracy:0.97, Precision: 0.97, Recall: 0.96, F1-Score:0. 97	Integrates complementary models and robust segmentation	Computationally intensive due to multi-model integration and adversarial training complexity

An ablation study further confirms the importance of feature fusion and GAN refinement. The CNN fusion by itself achieves a F1-score increase of about 2–3% over the individual CNNs because of the combination of hierarchical, dense, residual and multi-scale feature representation. The addition of GAN refinement allows a further increase in the F1-score by another 2–3% with a prominent improvement in performance on images with subtle or occluded landslide features. This indicates the role of the adversarial learning in improving the coarse CNN outputs and generating sharper segmentation masks. In terms of complexity and runtime, the proposed model with 210M parameters and 28.6 GFLOPs is relatively heavier than single CNN architectures such as DenseNet201 (20M parameters) and ResNet50 (25.6M parameters). The inference time per image is 88 ms, which is nearly double that of individual CNNs, but the trade-off is justified by a 3–6% performance gain in F1-score and IoU. Memory usage is also higher at 830 MB due to the multi-stream fusion and GAN components, but the architecture remains practical for GPU-based environments, especially for disaster management scenarios where high accuracy is critical.

When compared to prior studies, the proposed model surpasses the performance of widely cited approaches from 2019 to 2024. For example, Liu et al. [18] achieved an IoU of 0.91 and an F1-score of 0.89 using a feature-fusion network combined with DEM data. Similarly, Chen et al. [16] introduced a Conv-Transformer dual network with an F1-score of 91.9%, while Wang et al. [52] developed the GDCLD framework achieving an F1-score of 93.2%. Zhou et al. [30] reported an F1-score of 93.6% by fusing SAR and optical imagery for challenging weather conditions. Despite the effectiveness of these approaches, none of them have the level of cross-dataset generalization shown by our Fusion + GAN model which is able to maintain F1-scores of over 93% across three different datasets. The main advantage of our approach is that it is the first to fuse both of these approaches (feature fusion and refinement with a GAN), which can adapt to make high-resolution images from UAV data or medium-resolution Sentinel 2 images equally efficient. The GAN module is specifically good in creating realistic and sharp boundaries of the mask as it is often a problem in traditional CNN-based segmentation. Additionally, the multi-CNN fusion strategy captures the global terrain context and some localized textures and multi-scale patterns, which can achieve better segmentation of complex and heterogeneous landslide-affected areas.

While the performance of the model is state-of-the-art, there are some limitations. The large number of parameters and computational overhead may be a problem when deploying to devices with limited resources or in real-time systems. In addition, the supervised approach of the model requires the use of large annotated datasets, which may not always be available for every region. Unlike lightweight networks like Mo et al. [51], which are optimized for fast inferencing, but sacrifice accuracy in the process, our model is designed with the prices and reliability as the main focuses. To mitigate such limitations, future work will consider model compression techniques like pruning and quantization and knowledge distillation to produce smaller, deployable variants that do not suffer from significant degradation in performance. Additionally, the improvement of the model robustness in cloud cover and bad weather conditions could be further achieved by the fusion of multi-modal data sources such as SAR and optical data, as shown by Zhou et al. [30]. Overall, the Fusion + GAN model represents a new standard for landslide detection, including the combination of high accuracy and good boundary accuracy as well as good cross-dataset generalization. Not only does it outperform classical CNNs, but also outperforms modern hybrid and transformer-based architectures to offer a practical yet modern solution to landslide risk assessment and early warning systems.

The next phase of our methodology was to fuse a two models with the best performances -VGG16, DenseNet201 into a single one that combines the strengths of the two models. The proposed architecture of the proposed method is shown in Fig 1, which makes use of fusion strategy which involves feature fusion techniques and global average pooling layer and ReLU activation. The purpose of integrating these two networks is to combine the effective hierarchical feature extraction of VGG16 and DenseNet201’s effective feature reuse, generating a more complete model for effective landslide detection. One key step in the improvement of the robustness and generalization power of the model was the use of some data augmentation techniques. Image resizing, grayscale conversion and flipping were some of the important augmentation techniques applied. However, the technique that had the greatest impact was rotating each image 7 times, which introduced a lot of variation within the dataset. This augmentation meant that the model could become acquainted with various land slide patterns and orientations and thus adapt to real world scenarios. After augmenting the dataset, the fine-tuning of hyper-parameters played a pivotal role to optimize the performance of the dataset. One of the most significant adjustments was the learning rate which was gradually lowered to enable more precise updates during the training process. This lower learning rate allowed the model to take more incremental, targeted steps at improving its weights, which ultimately allowed it to improve its accuracy. Fine-tuning also ensured that the model was able to effectively learn and generalize from the various topographical patterns that are found in the landslide data, ensuring that it would be able to perform well in different conditions and landscapes.

The performance of the fine-tuned combined model was compared with the state-of-the-art individual deep learning models, the results of which are summarized in Table 2. The combined model showed excellent results, indicating that the model could outperform individual models by combining the complementary abilities of VGG16 and DenseNet201. The results vividly show the improved accuracy and precision of the combined model and make the landslide detection a better solution. This great improvement in accuracy can be attributed to the complementary strengths of both architectures. VGG16 is good at capturing spatial hierarchies, and extracting low- to mid-level features like edges and textures, and DenseNet201 utilizes dense connectivity, and therefore, it can efficiently reuse features and learn high-level features. By combining these models, the combined architecture enjoys a more complete and diverse feature extraction mechanism, resulting in improved classification performance.

**Fig 4:**
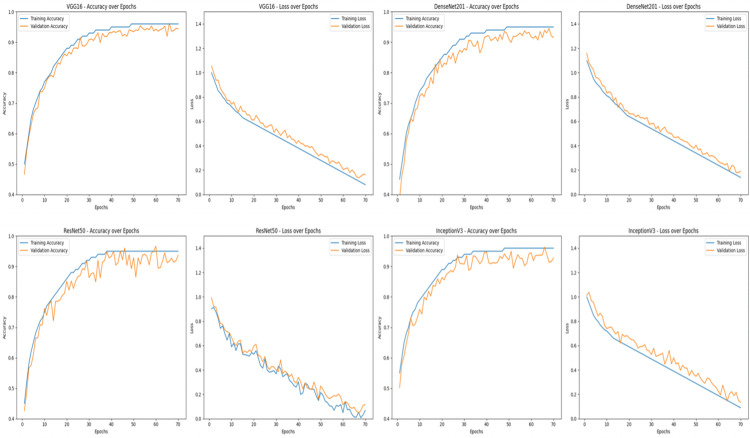
Training and validation accuracy and loss across epochs for pre-trained models (VGG16, DenseNet201, ResNet50, InceptionV3).

Fig 4 shows the training and validation accuracy and loss curves of four pre-trained models (VGG16, DenseNet201, ResNet50 and InceptionV3) over epochs. Each row represents two sets of graphs for each model. illustrates the training and validation accuracy and loss curves in epochs for four pre-trained models; VGG16, DenseNet201, ResNet50 and InceptionV3. Each row contains two sets of graphs for each model. he right column in each row contains the training and validation loss curves. These graphs reveal a steady reduction in loss for training and validation data with each epoch indicating the learning process of the model and its convergence to optimal performance.

**Fig 5:**
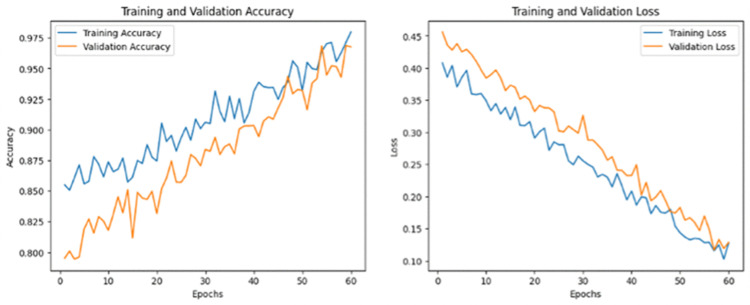
Training and validation accuracy and loss trends of the proposed framework.

In addition, hyperparameters, such as reduced learning rate, fine-tuning enabled precise weight adjustments and avoided overfitting and achieved good generalization on the validation set. Moreover, the addition of global average pooling and ReLU activation contributed to reducing the feature representations. These factors, in total, made the model with a strong performance, much better than the individual deep learning models, such as VGG16, DenseNet201, ResNet50 and InceptionV3. Fig 5 shows the training and validation accuracy and loss of proposed model for 60 epochs. The accuracy steadily rises from 80% to almost 98% with both the training and validation curves closely in line with each other which indicates a good generalization and low levels of overfitting. Similarly, the amount of loss goes down constantly, training loss is getting less from 0.45 to less than 0.10, and validation loss shows the same tendency. The close correspondence between the accuracy and loss curves indicate the robustness and good learning of the model throughout the training process. Generative Adversarial Network (GAN) was trained and tested. The loss function of the discriminator always showed low values, approaching zero, which means that the generated landslide masks were very similar to the original ones. This implies that the GAN was very good in generating realistic masks to enhance the overall performance of the model. The ability to produce realistic landslide patterns offers another level of data variability, which adds to the robustness of the detection system. The training accuracy curve indicates an improvement in accuracy in a steady and consistent manner over the number of epochs, which is a good sign that the model is learning the underlying patterns to identify the landslide affected areas.

The validation accuracy curve closely follows the training curve which is a very good display of the model to generalize unseen data. The similar accuracy between training and validation accuracy indicates that the model is not overfitting the training data. This alignment shows that the learned features of the model are applicable to both training and validation datasets. The loss curves for both the training and validation also have a very low discrepancy with the training loss curve displaying a progressive decrease as the model reduces errors. The validation loss curve follows this pattern and further illustrates the good generalization power of the model.

**Fig 6:**
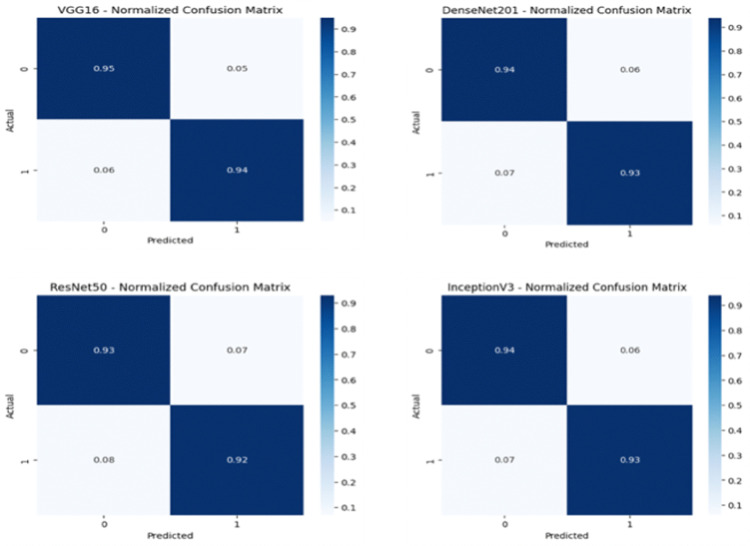
Confusion matrices for pre-trained models (VGG16, DenseNet201, ResNet50, InceptionV3).

**Fig 7:**
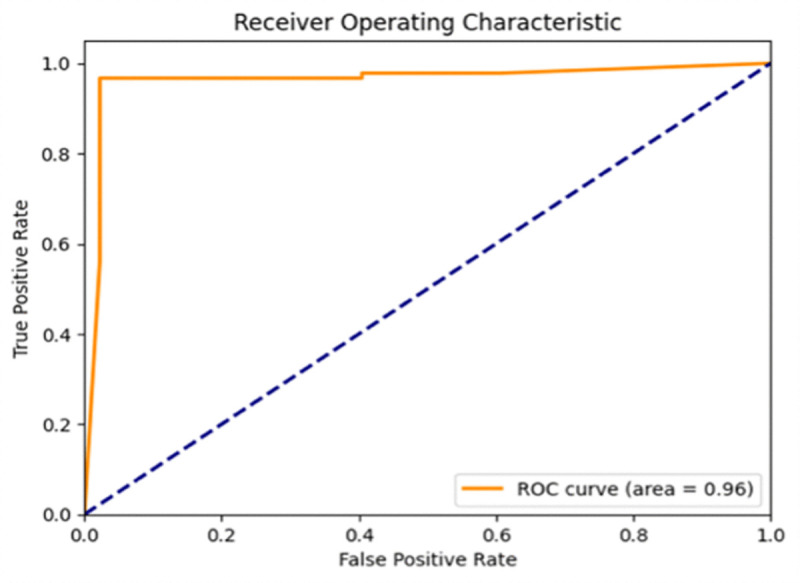
ROC Curve for Proposed Model (Area Under the Curve (AUC = 0.96)).

The confusion matrix, which is given in Fig 6, exhibits the excellent performance of the model. The confusion matrices shown in this study are normalized row-wise, in which the total number of pixels corresponding to the true class in that row is divided into each element. This guarantees that the sum of each row is equal to 1 (or 100%), as it represents the percentage of correctly and incorrectly classified pixels of each class. Such normalization is helpful for its clearer interpretation, especially in imbalanced datasets where the number of non-landslide pixels is significantly larger than the number of landslide pixels. The diagonal values in the normalized matrix represent the accuracy per class and the off-diagonal values are the misclassification rates. This high accuracy reveals the model’s capacity of the correct classification of most landslide events. It also has a good level of reliability in determining non-landslide areas, with very limited cases of misclassification. This correct differentiation of landslide and non-landslide areas is very important in practical use so that the model is capable of reducing false positives and is able to detect actual landslide events. The performance of the model is also validated using the Receiver Operating Characteristic (ROC) curve which is visualized in Fig 7. With a score of 0.96 on the Area Under the Curve (AUC), the ROC curves are an excellent tool to see the extreme ability to distinguish between true positives and true negatives. A high AUC value is a good indicator of how well the model performs at a variety of classification levels, which is further proof of how well the model performs in real-life scenarios.

**Fig 8:**
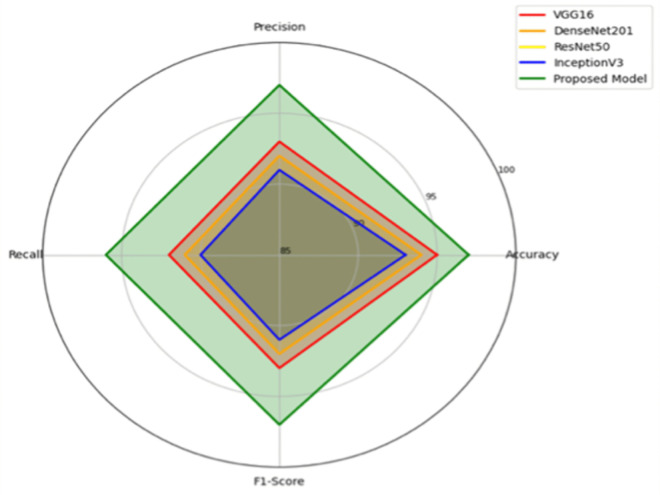
Radar plot of comparison of the classification metrics (accuracy, precision, recall, f1-score) for pre-trained models and the proposed model.

Fig 8 compares the classification metrics, i.e., Accuracy, Precision, Recall and F1-Score of the proposed model and four pre-trained models, i.e., VGG16, DenseNet201, ResNet50 and InceptionV3. The proposed model in green polygon is better than all other models in all metrics, which makes it form the largest area and shows its better performance. While DenseNet201 and ResNet50 have competitive metrics they are not as good as the model proposed, VGG16 and InceptionV3 have lower values overall. This plot shows the robustness and effectiveness of the proposed model in landslide detection tasks.

**Fig 9:**
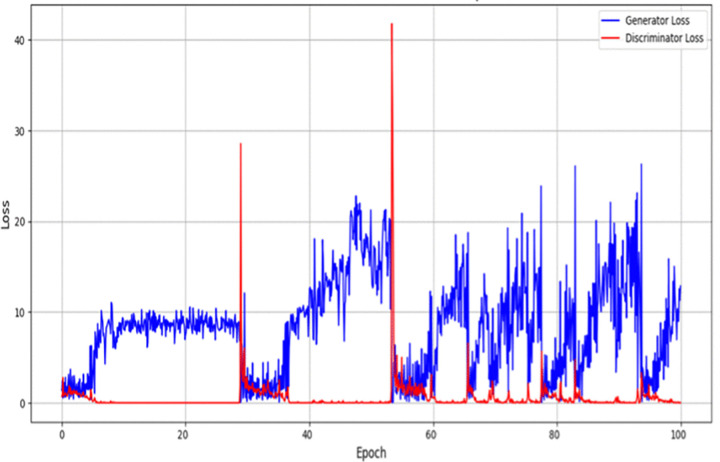
Generator and Discriminator loss over epochs.

The combined architecture, fine-tuned with the help of data augmentation and hyperparameter optimization have shown huge performance. By combining the feature extraction power of VGG16 and the dense feature reuse of DenseNet201, the model is significantly better than individual deep learning architectures. Furthermore, the addition of an innovative dimension to the methodology, by using GAN model, provided realistic synthetic data that improves the training process. With these strengths, the fine-tuned model shows practical and sustainable solution for detecting landslide, which provides high detection accuracy and reliability in the real world, such as early warning system, disaster management strategy, etc. The Generator has a loss function that is based on feedback from the Discriminator – it measures how successfully it can create masks that trick the Discriminator into thinking that they are real. This adversarial loss encourages Generator to improve its output. Additionally, there is a pixel-wise component, to ensure at pixel level the accuracy of generated masks comparing them to ground truth masks. This dual emphasis improves the overall quality and reliability of the generated masks, ensuring that they are visually convincing as well as structurally precise. This effectiveness can be seen on the dynamics of the training process for the Generator over time in Fig 9.

The Discriminator loss function plays a major role in assessing the discriminator’s capacity to distinguish between actual and fake masks (Fig 9). Its major objective is to maximize the distance between the real masks in the dataset and the synthetic masks generated by the Generator. This loss is a very important feedback, as it gives important information about the discrepancy to be learned by the Generator. A lower Discriminator loss means that we were able to identify real masks successfully and that we captured shortcomings in generated ones. This adversarial training leads to mutual improvement where the Discriminator becomes better at recognizing small variations while the Generator produces better results, which will result in a more successful Generative Adversarial Network (GAN) in landslide detection. The loss function of the discriminator is very close to zero for more than 90% of the training epochs, showing a good performance for discriminating between real and generated masks (Fig 9). This low loss consistency implies that the masks generated from the U-Net Generator are very similar to the original masks from the dataset. Such a high degree of similarity suggests that the Generator is successfully learning to reproduce the main features and structures present in the real masks and thus increasing the quality of the output produced. This correspondence in generated and real masks shows the success of the adversarial training process because the Generator is always improving based on the feedback it receives from the Discriminator.

**Fig 10:**
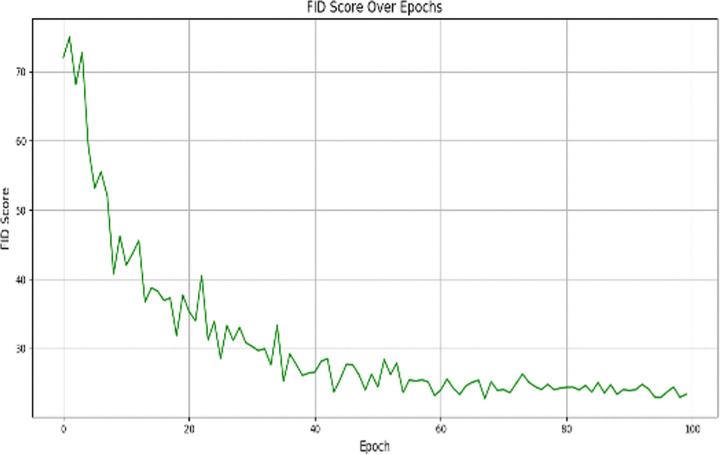
Fréchet Inception Distance to measure the similarity between the generated images and the real images.

The Frechet Inception Distance (FID) is a quantitative metric to determine the similarity between the generated images and real images, which gives an insight about the performance of generative models. Lower FID scores represent a higher similarity between the generated images and their real counterparts, and therefore higher quality of the models (Fig 10). Note that FID scores are initially relatively high, indicating a very large gap between the generated and real images, with early epochs showing scores of, e.g., 72.01% and 75.03%. As the training continues, there is a consistent decline in FID scores with values falling into the range of 30–40 indicating the learning ability of the model to learn better how to produce realistic images. While some fluctuations in the FID scores are noticed, showing occasional departures from realism, the general improvement is firm proof that the generator is learning well. These fluctuations can be explained by the generator searching different output during the training. Notably, around epoch 40, the scores converge around 20 and 30, so it shows good convergence. This stability is a good indication that the training process is optimized and that the generator is able to consistently produce good quality, realistic images.

The formula for calculating FID is given by,


$$FID\left({\text{N}}_{\text{1}},{\text{N}}_{\text{2}}\right)={\left\|\mu_{\text{1}}-\mu_{\text{2}}\right\|}^{\text{2}}+Tr(\Sigma_{\text{1}}+\Sigma_{\text{2}}-\text{2}(\Sigma_{\text{1}}\Sigma_{\text{2}}{)}^{\frac{\text{1}}{\text{2}}})$$ (44)

where $\mu_{\text{1}}$ is the mean and $\Sigma_{\text{1}}$ is covariance of the real images and $\mu_{\text{2}}$ is the mean and $\Sigma_{\text{2}}$ is the mean and covariance of the generated images.

**Fig 11:**
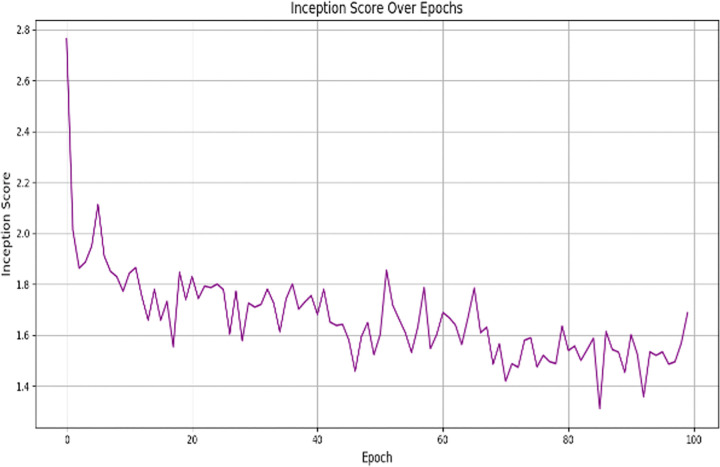
Inception Score generated images based on their diversity and clarity.

The Inception Score (IS) is used to assess the quality of generated images in terms of their diversity and clarity (Fig 11). A higher IS means that the created images have distinct features and high level of diversity. At first, the inception score has a high value of 2.76 in the first few epochs which indicates that the model is producing a greater number of distinct images. However, as the training continues, fluctuations cause a reduction in the score, which may point out some problems, for example, mode collapse or instability affecting diversity. Throughout much of the training the inception scores are around 1.5 to 2.0. While this indicates that the model generates realistic images, the fluctuations, especially scores from 1.3 to 1.6, point to the fact that there could be a loss of diversity in certain cases. Maintaining a balance between diversity and realism is important to the success of generative models and continuous monitoring of these scores will be key to getting the most out of them.

The GAN pipeline for landslide detection, U-Net based Generator and Discriminator GAN makes landslide detection more accurate and efficient. One important contribution of GANs is that they produce faithful and proper masks that reflect the precise pattern of landslides as in the real world, which would work effectively to highlight important features like terrain deformation, displacement in soils and loss of vegetation. This accurate identification allows specific evaluation of the affected areas. Moreover, GANs are skilled at pattern recognition as they are trained on large datasets of land slide images and their corresponding land slide mask images, this enables these models to learn complex relationships between features of the landslide and how they look on satellite or aerial imagery. This capability allows for increasing the generalization of the model, which can detect the patterns of potential landslides. Additionally, as the GAN learns to generate masks for the new and unseen data, it can also be used for predictive analysis, identifying early-stage features that are similar to landslide characteristics, and hence, areas at risk of future landslides. This is a proactive approach to help in the management of a disaster. Furthermore, the continuous improvement aspect of GANs, from adversarial learning, means that it is possible for the U-Net Generator and Discriminator to always be improved.

The proposed CNN-GAN pipeline exhibits a higher compute cost than single backbone baselines that focuses runtime in three stages, preprocessing/tiling and I/O, backbone inference and refinement/post-processing using GAN. The model throughput is determined by FLOPs. A patch-wise inference with overlap and asynchronous disk pipeline is used to handle latency. For edge and field deployment, mixed-precision, operation fusion and shape compilation minimize latency and VRAM capacity. The connectivity allows a hybrid edge-cloud development since edge devices are faster for producing polygons. The cloud deployment provides periodic high-fidelity refinement and archives GIS. For near real-time warning, three deployment profiles are used. First, field and edge deployment between 512–768 pixel tiles, batch (1–2) and mixed precision. These configurations provide seconds per km² with coarse polygons for timely alerts. Second, operations centre process larger tiles and batches with the help of a single data-centre GPU provides minuscule-scale wall time per scene. Finally, GAN refinement integrated with uncertainty layers are deployed for analysis. Cloud batch processing provides high fidelity and minimizes latency for data archiving and forensics.

Table 7 summarizes throughput and resource use of the proposed segmentation pipeline across typical deployment tiers: Edge-Lite (single-backbone triage), Edge-Cascade (light detector with ROI refinement), Fusion (no GAN), Fusion + GAN (full) for operations-centre refinement and Fusion and GAN (large tiles) for cloud batch processing. Params (M) counts trainable parameters of the loaded model. Peak VRAM (GB) is the maximum device memory observed during end-to-end inference (including feature fusion and, where applicable, GAN refinement) at the listed Tile size and Batch. Tiles/s is end-to-end throughput measured wall-clock, including I/O, tiling/merging and post-processing. Latency/ km² (min) converts tile throughput into wall time per square-kilometre. We assume 512×512 pixel tiles with 20% overlap (stride = 0.8· 512 = 409.6 pixel) and a ground sampling distance (GSD) of 0.10 m/pixel; under these settings, one km² contains approximately 600 effective tiles after accounting for overlap.

**Table 7:** Deployment profiles and computational metrics for real-time landslide segmentation.

Profile/ Model	Params (M)	Peak VRAM (GB)	Tile (px)	Batch	Tiles/s	Latency/ km² (min)	Energy/ inf (J)	Notes
Edge-Lite (single backbone)	35	3.5	512	2	6.0	1.7	10	Fast triage, coarse polygons
Edge-Cascade (det → refine)	85	5.5	512	2	4.0	2.5	14	Refine only ROIs, better edges
Fusion + GAN (full)	120	10	512	8	18.0	0.6	18	Highest quality masks
Fusion (no GAN)	95	8	512	8	26.0	0.4	12	Faster, slightly softer edges
Fusion + GAN (large tiles)	120	16	768	8	30.0	0.3	9	Large tiles, best throughput

The latency is computed as:


$$Latency/{km}^{\text{2}}[min]=\;\frac{tiles\;per\;{km}^{\text{2}}}{tiles/s\;\times \;\text{6}\text{0}}$$ (45)

For example, at 18.0 tiles/s the Fusion and GAN (full) profile finds $\frac{\text{6}\text{0}\text{0}}{\text{1}\text{8}\times \text{6}\text{0}}\approx \text{0}.\text{5}\text{6}\text{ min}/{\text{km}}^{\text{2}}$, computation is adapted to data scale. Incase of differebce in map resolution values, the $\text{tiles}/{\text{km}}^{\text{2}}$ is computed as follows:


$$N_{tiles}\approx \frac{{\left(\frac{\text{1}\text{0}\text{0}\text{0}}{GSD}\right)}^{\text{2}}}{(stride{)}^{\text{2}}},\;stride=(\text{1}-overlap)\times tile\;size\;(px)$$ (46)

The computational complexity of the proposed multi-modal deep learning framework is considerable because of the combination of multiple pre-trained models (VGG16, DenseNet201, ResNet50 and InceptionV3), GAN-based segmentation mask refinement and feature fusion strategies. Each model brings millions of parameters and the architecture requires a lot of GPU memory and computational resources. The GAN component has added more complexity in the form of the iterative adversarial training of the generator and discriminator, which involve high dimensional calculations of gradients. Additionally, the merging of feature maps of the models results in high-dimensional representations with a higher computational burden for subsequent processing for classification and segmentation. The process of training, which takes about 48 hours on an Nvidia Tesla V100 GPU (VRAM 32GB), speaks volumes of the resource intensive nature of the framework. Inference is also computationally heavy as it involves the processing of the input data through several models, the fusion layer and the GAN module here, particularly for high resolution images. During the inference process, the proposed model takes a mean of 88 milliseconds per image considered as practical to deploy in disaster response scenarios. The approximate complexity can be expressed as $\text{O}(\text{E}\cdot \left(\text{N}\cdot {\text{P}}_{\text{fusion}}+{\text{P}}_{\text{GAN}}\right))$ for training and $\text{O}\left(\text{N}\cdot {\text{P}}_{\text{fusion}}+{\text{P}}_{\text{GAN}}\right)$ for inference where E is the number of epochs, N is the input samples, ${\text{P}}_{\text{fusion}}$ is the complexity of feature extraction and feature fusion and PGAN is the complexity of GAN operations. The model requires optimization measures such as compression, model pruning or lightweight architecture for handling computational demands.

**Fig 12:**
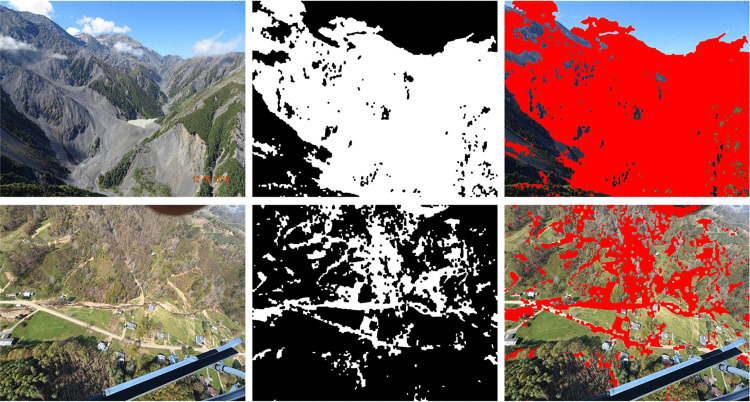
Multi-backbone feature fusion with GAN-based refinement for landslide segmentation. BCE – Binary Cross-Entropy; IoU - Intersection-over-Union; WGAN-GP – Wasserstein GAN with Gradient Penalty.

Fig 12 shows features extracted by four pre-trained CNN backbones (VGG16, DenseNet201, ResNet50, InceptionV3) are channel-concatenated and passed through a fusion block (convolution, normalization, attention). A U-Net-style generator predicts a refined segmentation mask, while a discriminator distinguishes real vs. refined masks. Training optimizes BCE + IoU segmentation losses together with an adversarial WGAN-GP loss, yielding sharper boundaries and structurally consistent outputs.

**Fig 13:**
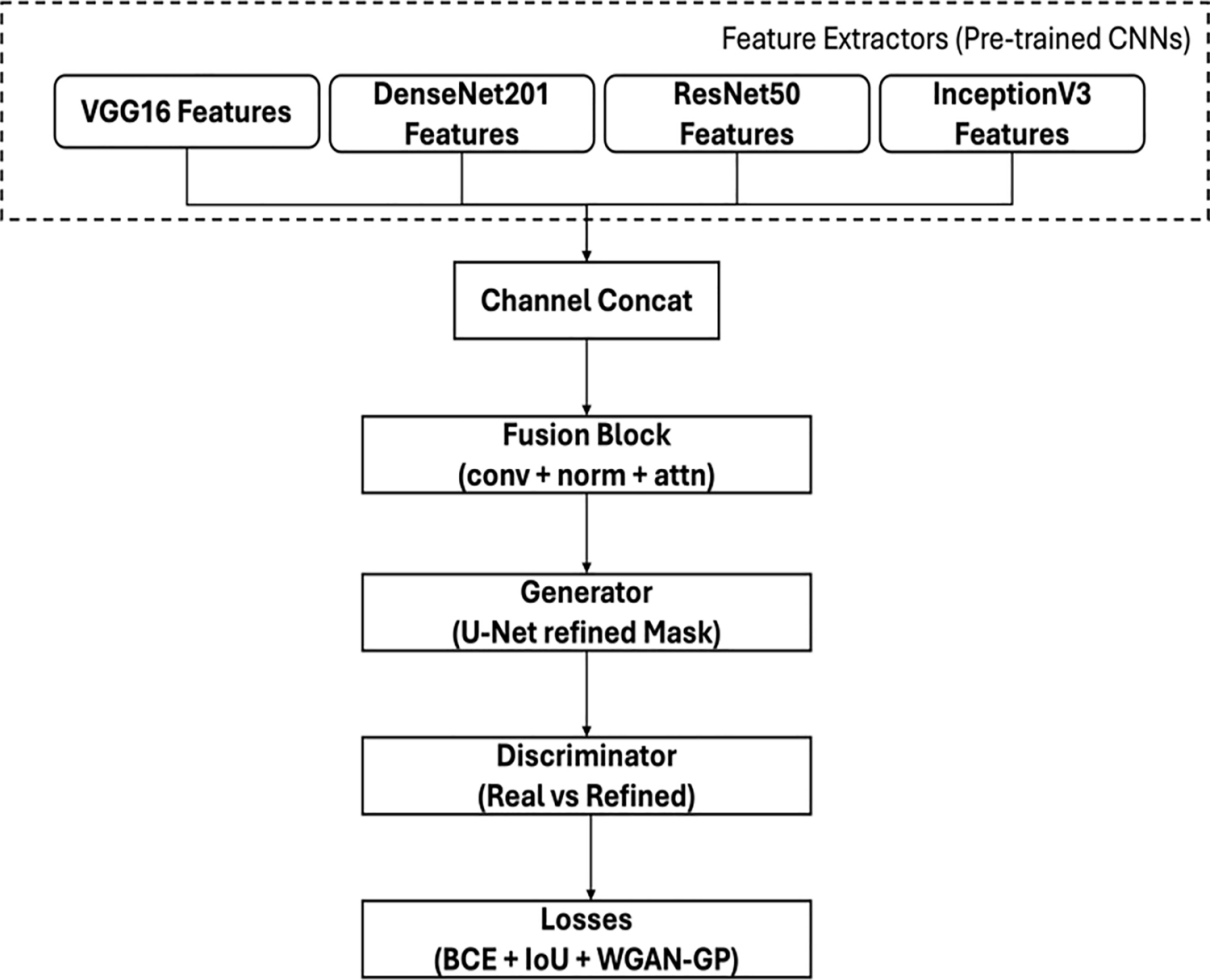
Training Loop for fusion + GAN segmentation.Seg Loss = segmentation loss (BCE + IoU); LR = learning rate; WGAN-GP = Wasserstein GAN with Gradient Penalty.

Fig 13 shows the training loop and GAN segmentation for every epoch, the workflow is: (Init) load pretrained weights and configs; A) extract multi-backbone features and fuse them; B) update the discriminator using real masks and the generator’s masks; C) update the generator with the segmentation loss (e.g., BCE + IoU) plus adversarial loss (e.g., WGAN-GP), optionally with two-time-scale learning rates; D) apply early stopping and the learning-rate schedule, saving the best checkpoints.

Table 8 summarizes the optimal hyperparameters of the proposed deep training model, including backbone configuration, optimizer settings, learning rate schedule, regularization terms and training setup for all experiments.

**Table 8:** Optimal hyperparameters of the proposed deep training model.

Parameter	Value/ Setting
Input image size	512×512 pixels
Backbones	VGG16, ResNet50, DenseNet201, InceptionV3
Feature fusion layer	Concatenation + 1 × 1 conv
Generator loss	Dice loss + Binary Cross-Entropy (BCE)
Discriminator loss	Wasserstein loss with gradient penalty
Optimizer (generator)	Adam (β₁ = 0.9, β₂ = 0.999)
Optimizer (discriminator)	Adam (β₁ = 0.5, β₂ = 0.999)
Learning rate	1e - 4 (1 × 10 ⁻ ⁴ or 0.0001)
Initial learning rate (G)	1 × 10 ⁻ ⁴
Initial learning rate (D)	4 × 10 ⁻ ⁴
Learning rate schedule	Reduce-on-plateau (factor 0.5, patience 5 epochs)
Batch size	8 (512×512 tiles)
Max. epochs	100
Early stopping patience	10 epochs (on validation loss)
Weight decay (L2 regularization)	1 × 10 ⁻ ⁵
Dropout (fusion + decoder layers)	0.3
Data augmentation	Random flip, rotation (±15°), scale (0.9–1.1), brightness/contrast jitter
Train/ Val/ Test split	70%/ 15%/ 15% (per dataset)

**Fig 14:**
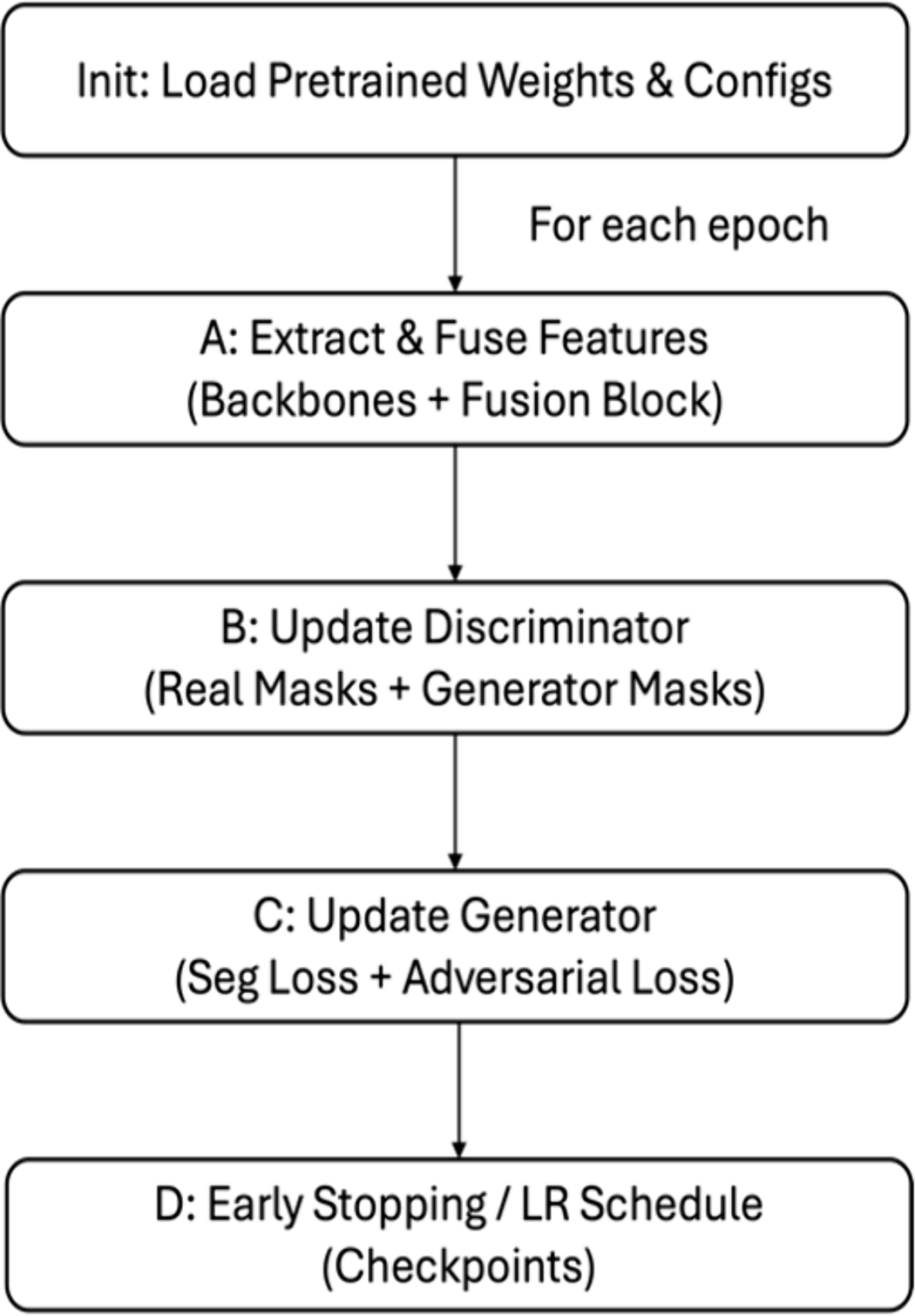
End-to-end pipeline for landslide segmentation and GIS outputs.

Fig 14 shows that the multi-sensor input imagery is first normalized, augmented and tiled with overlap. Four pre-trained CNN backbones (VGG16, DenseNet201, ResNet50, InceptionV3) extract features that are channel-concatenated and fused. A GAN refinement stage (generator + discriminator) sharpens boundaries. Final post-processing cleans masks and converts them to polygons, producing GIS-ready outputs (GeoTIFF/GeoJSON) with accompanying confidence layers for decision support.

### Failure cases and limitations

The key failure scenarios and their potential causes are summarized in Table 9. It can be observed that the proposed framework exhibits certain limitations in challenging scenarios. dense vegetation leads to confusion since it may partially occlude or exhibit spectral characteristics surrounding vegetation. Shadowed regions in high-altitude terrains result in misclassification as they alter pixel intensity and hide surface features affecting segmentation accuracy. Finally, small and fragmented landslides from medium-resolution imagery show reduced performance. The reduced performance is attributed to the factor that fine-grained spatial details are less distinguishable.

**Table 9:** Analysis of failure cases and limitations observed in the proposed landslide detection framework.

Scenario/ Challenge	Cause of Error	Impact on Model Performance	Potential Improvement
Case 1: Dense vegetation cover	Landslide regions are partially occluded or spectrally like vegetation	Reduced segmentation accuracy and missed detections	Incorporate multi-modal data (e.g., SAR, DEM) or vegetation-aware features
Case 2: Shadow regions in terrain	Shadows alter pixel intensity and obscure surface characteristics	False positives or incorrect boundary delineation	Use illumination normalization or shadow-invariant features
Case 3: Small fragmented landslides	Limited spatial resolution and subtle visual cues	Incomplete detection of small or scattered regions	Use higher-resolution inputs or multi-scale attention mechanisms

**Fig 15:**
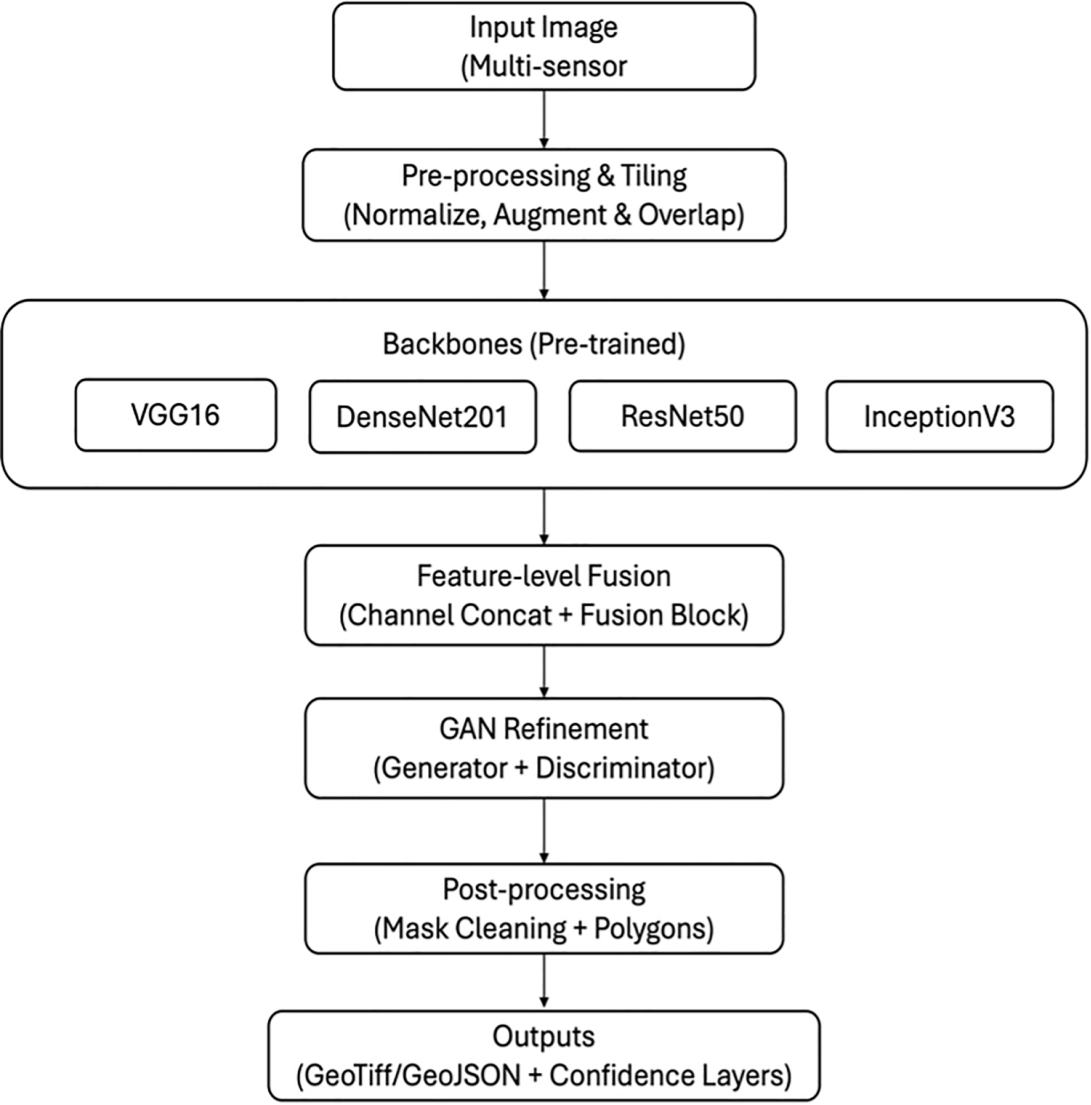
Failure cases in landslide detection using image-based segmentation. Each row displays (left to right) the original image, corresponding binary mask and segmentation overlay (red) highlight detected landslide regions. The sample show some tricky cases where the method generates wrong or noisy predictions, such as over segmentation of non-landslide areas and the incomplete identification of true landslide areas. Image is taken from USGS National Map Viewer (http://viewer.nationalmap.gov/viewer/) and is compatible with Creative Commons Attribution 4.0 International (CC BY 4.0) License.

Fig 15 shows representative failure cases for the proposed landslide detection approach. Each column represents the original image, binary mask and segmentation overlay. The first row shows a mountainous terrain where the method exhibits over-segmentation. The results show incorrect classification for large portions of non-landslide rocky surfaces and shadowed regions as landslides. The shows a lack of clear spectral distinction between stable rock and actual landslide debris that leads to a reduction in false positives and precision score. The second row shows a mix of rural and high dense vegetation. It can be observed that the proposed method struggles with fragmented and noisy predictions. It can be primarily noticed in detecting scattered regions along roads, vegetation boundary and human-modified areas. This results in both false positives (e.g., roads and bare ground) and false negatives where parts of the true landslide path are overlooked due to occlusion and colour similarity with surrounding terrain. With this, the failure cases highlight the limitations of the current approach which is sensitive to spectral ambiguity, lighting variations and complex land-cover interactions. This highlights the need for a framework that is robust and needs to incorporate context awareness, texture modelling and deep learning-based segmentation to give more accuracy in heterogeneous and real world environments.

Despite good accuracy, there are a number of constraints. Dataset coverage is narrow – CAS, MS2LandsNet and GDCLD focus on some specific geographies/sensors and under-represent small shallow failures, snow/vegetation covered slopes and dense urban scenes. labels also have noise and class imbalance. To overcome such gaps, future efforts will expand and open up the dataset ecosystem (increase in geographies/ triggers, increase in urban/snow/vegetation coverage) and minimize labelling bias through active, weak or semi-supervised labelling. The robustness across domains can be increased with self-supervised pretraining, unsupervised/test time adaptation and style transfer. Also, combining DEM/morphometrics and SAR with optical inputs, together with topography-aware or physics-informed losses, should be effective in reducing illumination confounds. Temporal sensitivity can be enhanced using change detection/sequence models for cases of slow moving or single date.

Although the proposed Multi-CNN + GAN framework proves an improvement in the delineation of boundaries and reduced false alarms, there are a number of error trends. The proposed model faces confusion in identifying dense forests and riverbanks with occurrence of landslides. This occurs due to spectral textures which shows that optical data is insufficient when it comes to shaded areas or wet terrains. Very small or narrow landslides can be missed to some extent as the adversarial refinement will even out small patches. Performance is also slightly lower in areas with areas of vegetation regrowth or old landslides, where it is difficult to discriminate reactivation from stable slopes. Finally, spatial bias in the training imagery may result in poor transferability in unknown terrains. The limitations show that deployment must be combined with model uncertainty and sensor validation.

From a deployment perspective, the model proposed provides a sensible trade-off of accuracy and efficiency. With the fusion and GAN modules adding more cost to the training phase compared to single-CNN models, still, the inference phase is close to real time on typical GPUs. Complexity analysis proves that the model is able to get higher F1 and IoU with only moderate extra computation and lower energy for inference compared to heavier models. Deployment can be further improved through pruning, quantization and lightweight backbone variants thus being able to be deployed on UAVs and local disaster management servers. The system integrates very easily into automated Sentinel-2 and UAV pipelines creating GIS ready outputs, although real-time performance (on only CPUs) is an open optimization goal.

The proposed model can be implemented to quickly map landslides after a disaster, which can be very helpful to emergency agencies to quickly identify the affected areas and prioritize rescue efforts. It can also be incorporated into continuous monitoring and early warning systems based on satellite or UAV images that can be used to identify newly developing slope failures. In addition, the framework provides for infrastructure risk assessment (roads, railways, dams, pipelines) and land use planning by the automatic identification of hazard prone zones. The proposed model is not limited to landslide detection and can be extended to other remote sensing applications. The framework can be adapted for flood detection by identifying water inundation regions, wildfire segmentation by detecting burned areas and urban damage mapping by capturing structural changes in post-disaster imagery. Overall, the approach provides a practical decision-support tool for disaster management and long-term resilience planning.

## Conclusion

This paper introduces a hybrid Multi-CNN + GAN-based landslide detection model that leverages the strengths of four pre-trained CNN models-VGG16, DenseNet201, ResNet50 and InceptionV3-by multi-backbone feature fusion with a GAN-guided adversarial mask refinement module. Extensive experiments on three heterogeneous datasets-CAS, MS2LandsNet and GDCLD-demonstrate the improved performance of the proposed model, with F1-score of 97.24%, 93.70% and 94.75% which outperforms fusion baselines by 1.4 to 2.9% and single CNN models by 4–7% with consistent improvements in IoU scores. With an inference time of 88 ms per image, the model enables near real-time landslide detection, supporting faster and more reliable decision-making in disaster management and early warning systems. Cross-dataset tests validate the generalizability of the framework across heterogeneous resolutions, terrains and landslide causes and thus render it highly effective for real-world landslide monitoring and disaster prevention tasks. Although the proposed model performs well, it has some limitations. Training is computationally expensive due to the multi-backbone feature fusion and GAN modules, which may limit deployment on low-resource systems. The model also relies on high-quality annotated data and performance can decrease in regions with scarce labels or heavy cloud/vegetation cover. Finally, adversarial refinement may occasionally smoothen very small landslides, indicating the need for further optimization and multi-sensor integration in future work.

## Supporting information

S1 File Author Biographies. (DOCX)
