# Supplementary material for: A multi-modal deep learning framework with GAN-based fusion for enhanced landslide detection
Source: PLoS One. 2026 Apr 30;21(4):e0347324. doi: 10.1371/journal.pone.0347324 (PMC13132460; doi:10.1371/journal.pone.0347324)
Supplement: S1 File — (DOCX) [file pone.0347324.s001.docx]

**Author Biographies**

- Deepika Roselind Johnson is working as an Assistant Professor (Senior Grade) in Vellore Institute of Technology, Chennai Campus. She completed her Ph.D in Computer Vision in 2023 and has over 10 years’ experience in teaching. Her major field of study includes Machine Learning, Internet of Things and Human Computer Interaction.
- Logeswari G is working as an Assistant Professor (Senior Grade) in Vellore Institute of Technology, Chennai Campus. She completed her Ph.D in Cloud Computing in 2024 and has over 8 years’ experience in teaching. Her major field of study includes Cloud Computing and Machine Learning.
- R Srivats is currently pursuing his B.Tech Computer Science and Engineering from Vellore Institute of Technology, Chennai Campus. He completed his schooling from PSBB Millennium, Chennai in 2021. His area of interest includes Computer Science, Machine Learning, Deep Learning, Machine Vision, Explainable AI and Blockchain.
- Saimirra R is currently pursuing her B.Tech Computer Science and Engineering from Vellore Institute of Technology, Chennai Campus. She completed her schooling from PSBB Millennium, Chennai in 2021. Her area of interest includes Artificial Intelligence, Machine Learning, Deep Learning, Explainable AI and Web Development.
- Muskaan Siddiqui Lucknow is currently pursuing her B.Tech Computer Science and Engineering from Vellore Institute of Technology, Chennai Campus. She completed her schooling from Spring Dale College, Lucknow, Uttar Pradesh 2021. Her area of interest includes Computer Science, Machine Learning, Full Stack Development and Cybersecurity.

Abhiram Sharma is currently pursuing his B.Tech Computer Science and Engineering with specialization in Data Science from Vellore Institute of Technology, Chennai Campus. His area of interest includes Artificial Intelligence, Financial Literacy and Technological Innovations. He is actively involved in interdisciplinary research projects across Healthcare, Agriculture, IoT, Cybersecurity and Financial Management.
